# Public perceptions of snakes and snakebite management: implications for conservation and human health in southern Nepal

**DOI:** 10.1186/s13002-016-0092-0

**Published:** 2016-06-02

**Authors:** Deb Prasad Pandey, Gita Subedi Pandey, Kamal Devkota, Matt Goode

**Affiliations:** Department of Herpetology, Senckenberg Research Institute and Natural History Museum, Senckenberg Biodiversity and Climate Research Centre, JW Goethe University, Frankfurt am Main, Germany; Institute for Social and Environmental Research, 57, Bharatpur, Fulbari, Chitwan, Nepal; Central Department of Zoology, Tribhuvan University, Kirtipur, Nepal; Wildlife Conservation and Management, School of Natural Resources and Environment, University of Arizona, Tucson, AZ USA

**Keywords:** Snake species diversity, Snake identification, Conservation, Snake worship, Snakebite, Ethno-ophiology, Ecosystem health, Key stone species

## Abstract

**Background:**

Venomous snakebite and its effects are a source of fear for people living in southern Nepal. As a result, people have developed a negative attitude towards snakes, which can lead to human-snake conflicts that result in killing of snakes. Attempting to kill snakes increases the risk of snakebite, and actual killing of snakes contributes to loss of biodiversity. Currently, snake populations in southern Nepal are thought to be declining, but more research is needed to evaluate the conservation status of snakes. Therefore, we assessed attitudes, knowledge, and awareness of snakes and snakebite by Chitwan National Park’s (CNP) buffer zone (BZ) inhabitants in an effort to better understand challenges to snake conservation and snakebite management. The results of this study have the potential to promote biodiversity conservation and increase human health in southern Nepal and beyond.

**Methods:**

We carried out face-to-face interviews of 150 randomly selected CNP BZ inhabitants, adopting a cross-sectional mixed research design and structured and semi-structured questionnaires from January–February 2013.

**Results:**

Results indicated that 43 % of respondents disliked snakes, 49 % would exterminate all venomous snakes, and 86 % feared snakes. Farmers were the most negative and teachers were the most ambivalent towards snakes. Respondents were generally unable to identify different snake species, and were almost completely unaware of the need of conserve snakes and how to prevent snakebites. Belief in a snake god, and the ability of snakes to absorb poisonous gases from the atmosphere were among many superstitions that appeared to predispose negativity towards snakes of BZ residents.

**Conclusion:**

People with predisposed negativity towards snakes were not proponents of snake conservation. Fear, negativity, ambivalence towards, and ignorance about, snakes and the need for snake conservation were strong indicators of the propensity to harm or kill snakes. It seems that if wanton killing of snakes continues, local snake populations will decline, and rare and endangered snake species may even become locally extirpated. Moreover, inappropriate perception and knowledge about snakes and snakebites may put BZ people at increased risk of venomous snakebite. Therefore, intensive, pragmatic educational efforts focused on natural history and ecology of snakes and prevention of snakebite should be undertaken in communities and at schools and universities.

## Background

Human attitudes towards snakes can be both positive and negative [1, 2]. In some places, people possess a deep respect for snakes due to spiritual traditions [3], while in other places people value snakes for utilitarian reasons [2, 4, 5]. However, snakes are typically misunderstood, mistreated, feared or killed, even when humans consider snakes to be symbols of power and worthy of worship worldwide [3, 6–8]. The consequences of negativity, ambivalence, fear, and killing of snakes for biodiversity conservation and human welfare have rarely been studied. Because snakes and snake parts are used in many different ways by different cultures, human activities can influence snake populations and communities both directly and indirectly. Therefore, snake-human interactions and the importance of ethnoherpetology [9] must be considered when planning conservation actions [10, 11].

A lack of knowledge and misguided perception of snakes threaten snake populations worldwide. Anthropogenic habitat fragmentation or destruction [12] and intentional killing of snakes [13, 14] contribute to snake population decline. If wanton killing of snakes goes unchecked, it will likely add to the risk of population decline, and even local extirpation of rare and endangered snake species, which may have cascading community- and ecosystem-level effects. In Nepal, the conservation status of snakes is either unknown or poorly defined based on minimal survey efforts carried out in the distant past, or simply confined to expert opinion [15]. Human activity, including intentional killing of snakes, likely contributes to population declines in many species, some of which play an important role in agricultural and grassland ecosystems of southern Nepal, which in turn may lead to negative impacts to biodiversity and human health. In addition to increasing our knowledge of snake ecology and natural history, it is important to assess public perception and knowledge of snakes. From a human health perspective, it is vitally important to better understand snakebite care and prevention among people inhabiting snakebite prone regions, which in turn represents a key component of snake diversity conservation, snakebite prevention, and prehospital care of snakebites.

Human and snake conflicts are commonplace throughout the world. People engaged in agricultural practices that utilize local resources from protected or non-protected areas for their living and sociocultural requirements, such as those living in the buffer zone of Chitwan National Park (BZCNP) in southern Nepal, suffer from life threatening snakebite envenoming. The threat of potentially fatal snakebite results in often ruthless killing of snakes. Therefore, it is important to understand the perceptions of rural villagers towards snakes, including assessing general knowledge about snakes, frequency and care of snakebites, and preventive measures taken. Armed with this knowledge, it is imperative to engage inhabitants in educational efforts that will lead to more appropriate responses towards snakes, which is expected to reduce snakebites and minimize life threatening interactions with snakes resulting in enhanced conservation of snake populations [16]. Although assessing attitudes and perceptions towards charismatic megafauna has been the subject of recent research [17] similar attention has not been given to assess attitudes, knowledge, and awareness of snakes and snakebite among people inhabiting BZCNP.

Snakes may be keystone predators [18, 19], especially in agricultural and grassland ecosystems, because snakes are effective predators of rodents. Indeed, snakes likely help to regulate food webs in important ways that other predators cannot. Snakes are also excellent ecological indicators due to their sensitivity to temperature and climate change [20]. Therefore, massive killing of snakes likely influences trophic interactions in ecosystems and may alter predator–prey population dynamics in multifaceted ways.

It seems reasonable to assume that high levels of human-caused mortality of snakes will result in an increase in rodent populations that will lead to a reduction in pre- and post-harvest cereal grains, other agricultural products, and household goods [21–25]. Increased rodent populations may also increase the risk of epidemic plague [26, 27] and diseases caused by *Salmonella* and *Campylobacter* [28]. Subsequently, snakes contribute directly to maintain natural trophic interactions, and indirectly to public health by reducing disease and famine. Although seemingly counterintuitive, unsustainable killing of snakes may also lead to increased snakebite [29], because individuals attempting to kill snakes are more likely to be bitten. Therefore, understanding causes of snake-human conflicts is essential.

Use of snakes for food, medicine, goods (e.g., snakeskin belts, purses, bags) and recreation (e.g., keeping snakes as pets, at zoos and for display by charmers) also threatens snakes. Worldwide, people use about 165 reptilian species, including snakes, for traditional medicine [10] and several ethnoherpetological studies indicate that traditional knowledge is important to herpetological conservation and human health [10, 30–33]. But similar studies are rare worldwide [34, 35], including in Nepal [36]. This study also highlights the human exploitation of snakes in BZCNP.

To escape from anthropogenic disturbances (e.g., forest fires, deforestation), natural predators in protected and non-protected forests, and flooding, snakes may retreat to human habitations, where they can find food (prey animals) and shelter, leading to a potential increase in snakebite envenoming, which can lead to death if not properly treated. It is not yet known how rural people react to snakes encountered in their homes compared to human-snake interactions that take place outdoors (e.g., roads, agricultural fields).

To the best of our knowledge, this is the first study to investigate perceptions, knowledge, and awareness (AKA) of snakes and snakebite in Nepal. Our goal is to provide baseline data useful for conservation of local snake populations and for enhancing snakebite prevention. To achieve this goal, we assess AKA by occupation, gender, and literacy of people living in BZCNP. We also determine challenges to snake conservation and snakebite management and provide insights and measures to improve AKA to address these challenges. This study informs major questions associated with anthropogenic threats to snakes and broad challenges to snakebite management [37]. Because teachers, students, and farmers are important for dissemination of conservation and public health education, quantifying their AKA may be of heightened importance for effective snake conservation and public health policy making.

## Methods

### Study area

A total of 35 BZ communities (15 from Chitwan, 16 from Nawalparasi, two from Makawanpur, and two from Parsa Districts [38]) surround CNP. Approximately 364,000 people inhabit these communities [39]. These people depend, both directly and indirectly, on resources found in the park and buffer zone areas. A rapidly increasing population in the Chitwan Valley has resulted in increased impacts on biodiversity and other natural resources in the vicinity. There is nearly an equal proportion of agricultural (46 %) and forested lands (50 %) in the BZ of CNP [38]. Agricultural lands are comprised of rice paddies, maize, and wheat fields, and forested lands consist of *Shorea robusta* (Sal) (30 %), tropical mixed hardwood (19 %) and *Accacia* species (Khair) and *Dalbergia sissoo* (Sissoo) (1 %) [38]. Both agricultural and forested lands appear to provide suitable habitat for snakes and their prey (e.g., rodents, birds, reptiles, amphibians and fishes) and predators (e.g., raptors, carnivores, other snakes).

The study area is characterized by a tropical climate, with temperatures up to 38 °C in summer, dropping to a minimum of 6 °C in winter, and receiving approximately 240 cm of rainfall annually, with the bulk occurring during the monsoon season [40].

**Fig. 1:**
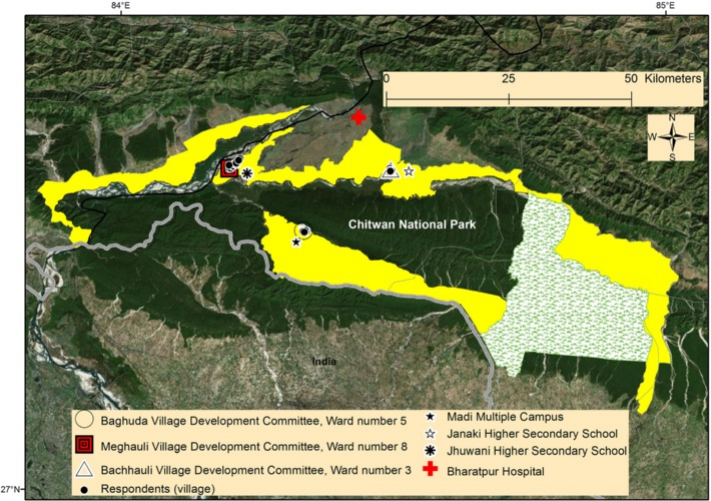
Map showing study sites in the southcentral lowlands of Nepal. Yellow highlighted area represents buffer zones of Chitwan National Park

We purposely selected three distantly distributed Village Development Committees (VDCs) adjoining CNP to represent a diverse array of BZ communities (Fig. 1). We selected three institutions of higher education and randomly selected three wards (i.e., the smallest administrative unit of Nepal). From these study units, we randomly sampled 75 household heads from a current household list of respective wards, and 45 teachers and 30 students from the daily attendance-register of each institution (Table 1).

**Table 1:** Study sites and study samples

Study sites	Sampling units (classes of listed educational institutions below, wards of Village Development Committee (VDC^a^ ))	Sampling unit population	Sample size (i.e. number of respondents)	Sample size (%)
Bachhayauli	Class 12, Jhuwani Higher Secondary School, Bachhayauli 09, Chitwan	33 (10 students, 23 teachers)^b^	25 (10 students, 15 teachers)	76
	Ward number 3, Bachhayauli VDC^a^, Chitwan	148 household heads^c^	25 (household heads)	17
Baghauda	Bachelor’s degree of Business Studies, 1st year, Madi Multiple Campus, Tribhuvan University, Baghauda 03, Chitwan	44 (24 students, 20 teachers)^b^	25 (10 students, 15 teachers)	57
	Ward number 5, Baghauda VDC^a^, Chitwan	87 household heads^c^	25 (household heads)	29
Meghauli	Class 11 and 12, Janaki Higher Secondary School, Telauli, Meghauli 05, Chitwan	60 (28 students, 32 teachers)^b^	25 (10 students, 15 teachers)	42
	Ward number 6, Meghauli VDC^a^, Chitwan	43 household heads^c^	25 (household heads)	58
	Total	415	150 (30 students, 45 teachers, 75 villagers)	36

Symbols: ^a^each VDC consists of nine wards which are the smallest administrative units of Nepal, ^b^obtained from daily attendance register, ^c^obtained from community forest register and social workers of respective wards, **%** (percent) = sample size / unit sample population x 100

### Data collection

**Fig. 2:**
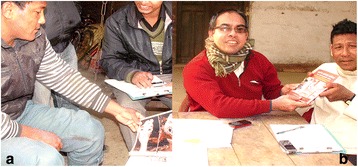
Displaying photo stimuli i.e. native snakes photos to informant (photo **a**) and providing non-monetary incentive i.e. Nepali medium snake related book after the interview (photo **b**)

We conducted a cross-sectional survey using semi-structured and pre-tested questionnaires, qualitative and quantitative research methods [41, 42] from January-February 2013. We performed personal, formal, and face-to-face interviews of 150 randomly selected respondents with a mean age of 37 years (range = 15–79) using a voice recording device and visual stimuli (i.e., A4-sized color photographs of adult snakes known to be distributed in the vicinity of CNP; Fig. 2, Table 2) [43]. We also included photographs of neonate and juvenile snakes for species with ontogenetic variation in color patterns.

**Table 2:** Checklist of snake photographs used while interview

PN	Scientific name	Common name	Vernacular name	Toxicity
	Typhlopidae			
1	*Indotyphlops braminus*	Brahminy Worm Snake/ Common Blind Snake/ Brahminy Blind Snake	Andha Sarpa or Sanp/ Ganeule Sanp/ Teliya Sarpa/ Nelia Sarp/ Matti Sanp/ Dhudh Sanp/ Andhara Sanp	Nv
2	*Argyrophis diardii*	Diard’s Worm/ Diard’s Blind Snake/ Large Worm/ Western Large Worm Snake/ Indochinese Blind Snake	Phusre Telia/ Andha Sap/ Matti Sanp/ Dhudh Sanp/ Andhara Sanp/ Ganeule Sanp/ Teliya Sanp or Sarpa	Nv
	Erycidae (Boidae)			
3	*Eryx johnii*	Red Sand Boa/ Brown Earth Boa/ John’s Sand Boa	Domukhe or Lide or Laxmi or Mate Sanp/ Lal Dhusar/ Duitauke Sarpa/ Jhataha	Nv
	Pythonidae			
4	*Python bivittatus*	Burmese Rock Python	Ajingar/ Ajgar/ Thulo Pate Ajinger/ Sonakatar	Nv
	Colubridae			
5	*Coelognathus helena*	Common Trinket Snake	Singare Sarpa (long-striped snake)/ Gahane Sap/ Male Sap	Nv^a^
6	*Coelognathus radiatus*	Copper-headed Trinket Snake/ Copperhead Trinket Snake/ Copperhead Racer	Singare Sarpa/ Ratothauke Gahane Sap	Nv^a^
7	*Dendrelaphis tristis*	Common Bronzeback Tree Snake	Sirish or Sirise (tree living)/ Siris Rukh Sanp/ Shipu/ Laudanga	Nv
8	*Lycodon aulicus*	Common Wolf Snake	Chichinde (gourd-shaped snake)/ Dhamiloo Sanp/ Buwase Sarp/ Sikhaphyancha/ Sikham Phyancha/ Sikhphyancha	Nv
9	*Lycodon jara*	Yellow-speckled Wolf Snake/ Twin-spotted Wolf Snake	Jor Thople Sikhaphyancha	Nv
10	*Oligodon arnensis*	Common/Banded Kukri Snake/ Russet Kukri Snake	Pate Khukuri Sap/ Gurbay/ Pate Sikhan Pyancha/ Sankhad Sanp	Nv
11	*Oligodon kheriensis*	Coral Red Kukri Snake/ Coral Kukri Snake	Puwale Khukuri Sap/ Harrama (Rai community)	Nv
12	*Ptyas mucosa*	Asiatic Rat Snake/ Indian Rat Snake/ Indian Wolf Snake	Dhamin or Dhaman (big garlands), Dhamila or Dhamala/ Muse Sarpa/ Lambaiya (lanky snake)/ Bichhar (nipple sucking snake)	Nv
14	*Xenochrophis piscator*	Checkered Keelback	Pani Sarpa or Pani Sanp or Pani Syap (water snake)/ Kothe Dhodiya Sap/ Dhodiya Sanp/ Pankhadar/ Gareha Sarpa/ Dom	Mv, Vs
15	*Ahaetulla nasuta*	Common Vine Snake/ Common Green Whip Snake/ Green Vine Snake	Sugia or Suga Sarpa (parrot like or parrot snake)/ Hario Chabuke Sarpa/ Udne Hareu/ Harahara	Mv, Bf
16	*Amphiesma stolatum*	Striped Keelback/ Buff-striped Keelback	Bagale/ Nauri/ Nauria/ Ashare/ Harara/ Harihara/ Bahune Sarpa/ Harhare Sarpa/ Hurra/ Chyarra/ Dirisarp/ Deri/ Dondaha	Mv, Bf
17	*Boiga trigonata*	Common Cat Snake/ Indian Cat Snake/ Indian Gamma Snake	Sanbe or Sabhe (cylindrical snake, in Kirat or Limbu)/ Adhoo Sarpa/ Tirish/ Batashe Sarpa (windy or gliding snake)/ Bharati Birale Sap/ Basara (nesting snake)/ Lohagin (irony)/ Birale Sarpa (catlike snake)/ Batyoudesyaap (gliding snake)/ Chittar (cupid)/ Chudeu (crested)/ Katakhor (cutter of pen)	Mv, Bf
18	*Rhabdophis subminiatus*	Red-necked Keelback	Lal Kanthe Daline Sap	V, Vs
	Homalopsidae			
13	*Ferania sieboldi*	Siebold’s Smooth-scale Water Snake/ Siebold’s Smooth Water Snake	Dhod or Dhodia Sarpa/ Machhagidhi/ Chile Pani Sap/ Pani Sarpa	Mv, Bf
	Elapidae			
19	*Bungarus caeruleus*	Common Krait/ Common Indian Krait	Bairi Karet/ Kret Sarpa (file snake)/ Chure Karet/ Seto-kalo-chure Krait/ Ganaich/ Gadainch/ Ghod Gadainch (horse like krait)/ Kalaich (killing monster)/ Karkat nag (cancer snake)	V
20	*Bungarus fasciatus*	Banded Krait	Panhelo-kalo-chure Sarpa/ Kanthmala Sap (snake with necklace or garland)/ Laxmi Sarpa (money making snake)/ Ganguwali or Pate Ganguwali Sarpa/ Gangwari (cowshed living)/ Gun Gawari/ Gangwar/ Ganguri Sarpa/ Maher/ Gwala Sarpa (cow-herd snake)/ Rajasarp (king snake)/ Ahiriniyasarp (not looking snake)	V
21	*Bungarus lividus*	Lesser Black Krait	Kalo Krait (black krait)	V
22	*Sinumicrurus m. univirgatus*	MaClelland’s Coral Snake	Setofetawal Nag/ Muga Sanp/ Rato Sarpa/ Karkat Nag (cancer cobra)/ Nag/ Naag (semi-divine serpent)	V
23	*Naja kaouthia*	Monocled Cobra/ Monocellate Cobra	Goman/ Nag/ Ek Thople Goman/ Seto Goman/ Paniadarad (water burning pain)/ Supailyasyaap/ Tilakdom (with black hood marking)/ Dom/ Dumini	V
24	*Naja naja*	Spectacled/ Common Cobra	Goman (cobra, aggresive snake)/ Nag/ Dui Thople Goman/ Kalo Goman/ Dudhiya Goman (milky cobra)/ Dumini (female sweeper)/ Supailyasyap/ Supailesyap/ Supya Sarpa/ Phetara (expanded hood)/ Kopre (hooded or bent ahead)	V
25	*Ophiophagus hannah*	King Cobra	Queta or Kenwata/ Raj Goman/ Darad (much poisonous or paining)/ Nagraja (snake king)/ Alhaad (Sanskrit: fireband)/ Kalinag (black cobra)/ Bhainsedom (buffalo sweeper)	V
	Viperidae			
26	*Trimeresurus albolabris*	White-lipped Green Pit-viper/ White-lipped Bamboo Viper	Harau/ Harau Sanp/ Haryousarpa/ Setojibre Hareu Sap/ Pattar	V
27	*Daboia russelii*	Russel’s Viper	Baghe Sarpa, Suskar	V
28	*Echis carinatus*	Saw-scaled Viper	Karaute Sarpa	V

*Abbreviations*: *PN* photo number (PN 27 and 28 are presumed to be distributed in Chitwan valley and lowlands of Nepal (Shah and Tiwari 2004, Shrestha 2001). So, we included them despite these were not reported from Chitwan valley (Pandey 2012)), *Nv* Non-venomous, *Mv* Mildly venomous, *Bf* Back-fanged, *Vs* Venomous secretion, *V* Venomous; this checklist was adopted from: Pandey 2012, Shah and Tiwari 2004, Schleich and Kästle 2002, Shrestha 2001, Zug and Mitschel 1995). Although *Coelognathus radiatus* possesses postsynaptic neurotoxin in its Duvernoy’s gland (Fry et al. 2003), Harris et al. (2010) reported four *Coelognathus radiatus* bites on the feet causing pain and bleeding at the bite site. Therefore, I considered both nonvenomous snakes while analysing knowledge of locals on surrounding venomous snakes

Of the total number of people surveyed, 33 % were farmers^1^ (*n* = 50), 30 % were teachers (*n* = 45), 23 % were students (*n* = 34) and 14 % were classified as “other” (*n* = 21). Respondents were illiterate (20 %) to highly literate (32 %). Three respondents refused to share their education status. The literate respondents (80 %, *n* = 120) attained up to class 10 (21 %, *n* = 31), class 11–12 or equivalent intermediate degree (21 %, *n* = 31), and Bachelor’s and Master’s degree (32 %, *n* = 48). We surveyed 68 % males (*n* = 102, males and females ratio = 213) and 98 % Hindus.

Written informed consent was obtained from the participants for publication of this study and any accompanying images. For the informed consent, we clearly explained the main objectives of our research at the beginning of the interview and asked them if they would participate in the survey research. As for institutional respondents, we interviewed them after a formal request for permission to the principals of the respective institutions. We did not obligate any respondents to participate in this study.

### Attitudes

We asked 15 questions designed to understand positive attitudes and 14 questions designed to examine negative attitudes towards snakes and snake conservation. We determined ambivalent attitudes if participants responded “yes” to both types of questions. To scrutinize and measure attitudes, we asked participants questions related to like, dislike or fear of snakes, intention of killing snakes, responses to snakes encountered in defined and undefined places, worship of snakes, realizing the need of snake conservation, and snakes as a “farmers’ friend.” We phrased the first type of question as, *Do you …? Why?*; we coded responses as 1 = Yes, 2 = No, 3 = Unknown, and we noted three types of logic for Yes or No responses. We phrased the second type of question as, *What do you do when …?*; we coded responses as 1 = I ignore it, 2 = I kill it, 3 = I call others to kill it, 4 = I kill it only if I know it a venomous snake, 5 = I just keep it out using sticks (snake hooks, tong, etc.). We phrased the third type of question as, *Which of the following do you consider to be…?*; we coded responses as 1 = All snakes around us should be killed, 2 = Only venomous snakes around us should be killed, 3 = All snakes around us should be conserved. Again, we coded responses as 1 = Yes, 2 = No, 3 = Unknown.

### Knowledge

The knowledge test questions included three types of questions: the first type tested whether or not people could identify the snake as venomous or non-venomous, and if they knew the local/English/scientific names of the snake; second, we tested their understanding of the need for snake conservation; and third, we asked about measures of snakebite prevention. We presented the first type of question as, *Which one of the following snakes do you think were venomous or non-venomous?* (we considered both rear- and front-fanged snakes as venomous) and which snake species do you know by their local/English/scientific names?

To measure knowledge of the need to conserve snakes, we phrased questions such as, *Do you think snakes should be conserved?* and *If you do/don’t think so, why?* We asked respondents to give five reasons. To measure knowledge about snakebite prevention, we phrased questions such as, *Do you know how to prevent snakebite?* If yes, we asked them to give 10 preventive measures that they practice. We encircled the corresponding assigned snake photo numbers (i.e., 1–28) following their responses and noted names of respective snakes if they were able to identify the species. We crosschecked their replies with a corresponding list of snakes (Table 2) and published sources [44] during data entry.

### Awareness

To examine the snake awareness level among BZCNP residents, we asked 33 “yes-no” questions, which included both useful and useless, deleterious, and fictitious aspects of snakes and snakebite management [45]. Of the 33 questions, 26 were designed to test belief in popular, deep-rooted, and widely-held traditional beliefs or misconceptions regarding snakes (*n* = 13) and pre-hospital care of snakebites (*n* = 13). Two questions tested belief in doubtful benefits of pre-hospital care in the context of Nepal [45, 46], and five questions were related to first aid measures (i.e., pressure immobilization bandaging (PIB) and local compression pad immobilization (LCPI)) recommended by the World Health Organization and the Government of Nepal [44, 47–49].

To better understand ethno-ophiological issues, we asked respondents whether or not they or their neighbor killed snakes for food or ethno-medicine during the 1-year period of this study.

### Data analysis

We analysed composite AKA scores using the non-parametric Wilcoxon test with median scores as the dependent measure [50]. We used the one-sample Wilcoxon signed rank test to understand median scores for each demographic group for attitudes and knowledge, a two-tailed unpaired Wilcoxon rank sum tests to compare differences of scores among demographic groups, and a one-tailed unpaired Wilcoxon rank sum test to compare maximum scores among demographic groups. We did not conduct demographic-group analyses for sample sizes lower than six to minimize problems associated with measurement error.

We analysed awareness based on the percentage of median scores of respondents after conducting the Wilcoxon test. We classified BZCNP residents as “highly aware” (HA), if they scored ≥75 %, indicating rejection of traditional beliefs of snakes and snakebite care, doubt about refusing to seek medical attention for snakebites, and acceptance of suggested measures of pre-hospital care. Similarly, we considered respondents as “aware” (A) if they scored 50–74 %, “mildly aware” (MA) if they scored 25–49 %, and “unaware” (UA) if they scored 0–24 %.

We considered all tests to be significant at α = 0.05. We rounded *p*-values (*p*) to significant digits (values less than three significant digits were represented as *p* = < 0.001). We performed all analyses using the R statistical package (R version 2.15.1).

## Results

### Attitudes

**Fig. 3:**
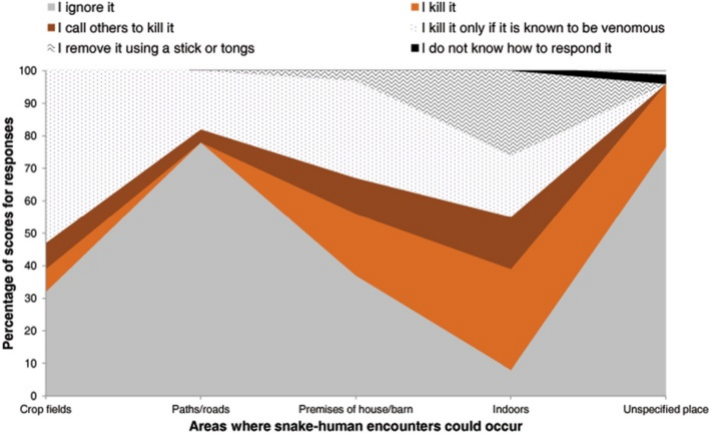
Human responses to snakes encountered in specified and unspecified locations in the buffer zones of Chitwan National Park

Residents of CNPBZ had higher scores for positive attitudes than for negative and ambivalent attitudes towards snakes and snake conservation issues. More than 47 % of respondents (*n* = 70) had positive attitude scores (median = 99, *p* = 0.047, Table 3) based on answering >8/15 questions (median = 9, *p* = <0.001, Table 4.a). Students, teachers, and literate respondents were more positive (Table 5.i). Positivity was not significantly different between males and females (*p* = 0.213, Table 5). Respondents had a positive temperament towards snakes in unspecified areas and areas with less human activity. Respondents generally ignored snakes encountered while walking on paths and 77 % remained tolerant to snakes at unspecified localities (Table 6.a, b, Fig. 3).

**Table 3:** Chitwan National Park buffer zone population with positive, negative and ambivalent attitudes to snakes

Hypothesis tests (for all respondents with different responses to attitude test questions, please, see questions in Table 6)	Median, range	Mean ± SEM	sd	W (res)	*p*-value	95 % CI
a. With positive attitudes (*n* = 15, see Table 6 .a); H0: M = M0 (70), Ha: M > M0 (70)	99, 12–148	91.6 ± 11.08	42.92	90	0.047	70.5–Inf
b. With negative attitudes (*n* = 14, Table 6 .b); H0: M = M0 (9), Ha: M > M0 (9)	13, 0–129	28.86 ± 9.84	36.82	81.5	0.037	8.5–Inf
c. With ambivalent attitudes (*n* = 9, see Table 6 .c); H0: M = M0 (14), Ha: M > M0 (14)	22, 7–62	28.11 ± 6.78	20.33	31	0.040	14.5–Inf

*Abbreviations*: *n* sample size i.e. total number of attitude test questions, *SEM* standard error of mean, *sd* standard deviation, *W(res)* value for one-tailed one-sample Wilcoxon signed rank test of respondents with attitudes (Table 6) to snake and their conservation, *CI* confidence interval, *H0* null hypothesis, *Ha* alternative hypothesis, *M* population median, *M0* hypothesized median

**Table 4:** Scores for attitudes of Chitwan National Park buffer zone people to snakes and their conservation

Demographics		a. Score for positive attitudes (*n* = 15, see Table 6.a) (null hypothesis (H0): population median scores (M) = hypothesized median scores (M0 = 8 of 15); alternative hypothesis (Ha): M > M0)			b. Scores for negative attitudes (*n* = 14, see Table 6.b) (null hypothesis (H0): population median scores (M) = hypothesized median scores (M0 = 2 of 14), alternative hypothesis (Ha): M > M0)			c. Scores for ambivalent attitudes (*n* = 9, see Table 6.c) (null hypothesis (H0): population median scores (M) = hypothesized median scores (M0 = 1 of 9); alternative hypothesis (Ha): M > M0)		
		Median, range	W (pos)	*p*-value	Median, range	W (neg)	*p*-value	Median, range	W (amb)	*p*-value
All respondents (150)		9,4–14	67885.5	<0.001	2,0–7	5058	<0.001	2,0–7	4618	<0.001
Age (years)	15–24 (42)	10,4–14	639.5	<0.001	2,0–6	332.5	0.044	2,0–5	337	0.001
	25–34 (22)	9,4–14	115	0.102	2,0–6	125	0.226	1,0–4	60	0.048
	35–44 (40)	9,5–13	478.5	0.001	2.5,0–7	336	0.037	2,0–7	357	0.004
	45–54 (21)	10,5–13	156	0.007	2,0–7	80	0.442	1,0–6	92.5	0.030
	55–64 (17)	9,6–13	107.5	0.021	3,1–6	104	0.006	2,1–5	120	<0.001
	65+above (8)	6,5–12	7	*0.901*	3,1–7	25.5	0.029	1,0–5	12	0.412
Gender	Male (102)	9,4–14	3317.5	<0.001	2,0–7	2130	0.055	2,0–7	2475.5	<0.001
	Female (48)	9,4–13	620	0.017	3,1–7	604	<0.001	1,0–5	335	0.012
Occupation	Farmer (50)	8,4–13	538.5	0.214	3,1–7	736	<0.001	1.5,0–6	490	0.001
	Teacher (45)	9,6–13	718.5	<0.001	2,0–6	401.5	0.321	2,0–7	451	<0.001
	Student (34)	10,4–14	414.5	0.001	2,0–6	185	0.269	1.5,0–5	197	0.002
	Other ^a^ (21)	10,6–14	158	0.001	2,0–6	91	0.411	2,0–4	129	0.025
Educational status	*Illiterate (27)*	8,5–13	143.5	0.439	3,1–7	211	0.003	1,0–5	115.5	0.026
	*Literate (120)*	9,4–14	4671	<0.001	2,0–7	3055.5	0.006	2,0–7	3212	<0.001
	≤ Class 10 (31)	8,4–14	195	0.193	3,0–7	329	<0.001	2,0–6	275	0.001
	Class 11–12 (31)	11,5–14	465.5	<0.001	1,0–6	136	0.918	2,0–4	190	0.012
	Master’s D (22)	9,6–13	238.5	0.006	2.5,0–6	153	0.186	2,0–7	170	0.001
	Bachelor’s D (26)	9.5,4–13	117	0.028	2,1–6	67	0.067	2,0–5	95	0.003
	Lit. inf. ^b^ (10)	11,5–13	49	0.015	1.5,0–6	17	0.584	0.5,0–3	18.5	0.5

Symbols and *abbreviations*: ^a^hotel owner (3), miller (3), fisherman (2), boat-man (1), mason (1), labourer (1), housewife (7), nature guide (3); ^b^respondents able to read and write by informal education but never attained school, < less than, > greater than, *n* sample size i.e. total number of attitude test questions, *D* degree, *W* value for one-tailed one-sample Wilcoxon signed rank test, *pos* positive, *neg* negative, *amb* ambivalent; parentheses in column demographics show number of respondents involved in statistical analysis

**Table 5:** Attitudes to and awareness of native snakes in Chitwan National Park buffer zone people

Demographics	a. Scores gained for attitude test						b. Scores gained for awareness test			
	i. Positive attitude		ii. Negative attitude		iii. Ambivalent attitude		i. Awareness		ii. Unawareness	
	W	*p*-value	W	*p*-value	W	*p*-value	W	*p*-value	W	*p*-value
Younger & older^a^ (Ha.1)	1168.5	0.743	1137.5	0.581	954.5	0.061	948	0.064	1390	0.229
Male & female (Ha.1)	2755	0.213	1836.5	0.012	2929	0.045	3447	<0.001	1876.5	0.021
Female > male (Ha.2)	x	x	3059.5	0.006	x	x	x	x	3019.5	0.011
Male > female (Ha.2)	x	x	x	x	2929	0.022	3447	<0.001	x	x
Farmer & student (Ha.1)	582.5	0.014	1182	0.002	824	0.808	623.5	0.039	795	0.618
Student > farmer (Ha.2)	1117.5	0.007	518	0.002	x	x	1076.5	0.020	x	x
Farmer & teacher (Ha.1)	793.5	0.013	1557.5	0.001	1019.5	0.418	412	<0.001	1410	0.033
Teacher > farmer (Ha.2)	1456.5	0.006	x	x	x	x	1838	<0.001	x	x
Farmer > teacher (Ha.2)	x	x	1557.5	0.001	x	x	x	x	1410	0.017
Teacher & student (Ha.1)	711.5	0.595	777.5	0.902	711.5	0.585	289.5	<0.001	1098.5	0.001
Teacher > student (Ha.2)	x	x	x	x	x	x	1240.5	<0.001	x	x
Student > teacher (Ha.2)	x	x	x	x	x	x	x	x	1098.5	<0.001
Literate & illiterate (Ha.1)	2128	0.011	1244	0.055	1798.5	0.356	2550	<0.001	1276	0.085
Literate > illiterate (Ha.2)	2128	0.005	x	x	x	x	2550	<0.001	x	x
Illiterate > literate (Ha.2)	x	x	1996	0.028	x	x	x	x	x	x

Symbols and *abbreviations*: ^a^15–34 years old people are considered young and 45–64 year respondents as older; *null hypothesis (H0): population median score (M)* hypothesised population score (M0 = 0), *alternative hypothesis.1 (Ha.1)* population median score (M) ≠ hypothesised population score (M0), *alternative hypothesis.2 (Ha.2)* population median score (M) > hypothesised population score (M0), *W* value for one- and two-tailed unpaired Wilcoxon rank sum test

**Table 6:** Responses of Chitwan National Park buffer zone people to attitude test questions (*n* = 38) about snakes and their conservation

SN	a. Responses to positive attitude test questions (*n* = 15); *note 1*: number of respondents with *unknown* reply to like or dislike of snakes were 4 (3 %), worship of snakes 1 (1 %), respond snakes that they encountered wherever and whenever 4 (3 %), friendly association between snakes and farmers 21 (14 %), need of conservation of all snakes 41 (27 %), all surrounding snakes should be killed 6 (4 %), and only venomous snakes should be killed were 4 (3 %).	Respondents	
		N	%
1	Yes, I like snakes	82	55
2	No, I do not fear snakes	21	14
3	Yes, I ignore whatever snakes I observe in the crop-field while working	79	53
4	Yes, I ignore whatever snakes I observe on the path while walking	117	78
5	Yes, I ignore whatever snakes I observe in premises of house or barn	55	37
6	Yes, I ignore whatever snakes I observe indoors	12	8
7	Yes, I rescue whatever snakes I observe indoors	39	26
8	Yes, I worship snakes	126	84
9	No, I do not prefer to kill whatever snakes I encounter anywhere	115	77
10	No, I do not eat snake meat	148	99
11	No, my neighbours do not eat snake-meat	139	93
12	No, my neighbours do not kill snakes even for medicinal purposes	129	86
13	Yes, all snakes around us should be conserved	99	66
14	Yes, I consider snakes as friends of farmers	92	61
15	Yes, I think snakes need to be conserved	122	81
	b. Responses to negative attitude test questions (*n* = 14); *note 2*: number of respondents having item non-responses for killing snakes wherever and whenever that they encounter were 2 (1 %), all surrounding snakes should be killed 77 (51 %) and only venomous snakes should be killed were 39 (26 %).		
1	No, I do not like snakes	64	43
2	Yes, I fear snakes	129	86
3	Yes, I kill whatever snakes I observe in crop-field while working	10	7
4	Yes, I call others to kill whatever snakes I observe in crop field while working	12	8
5	Yes, I kill whatever snakes I observe on the path while walking	0	0
6	Yes, I call others to kill whatever snakes I observe on the path while walking	6	4
7	Yes, I prefer to kill whatever snakes I encounter anywhere	29	19
8	Yes, I eat snake meat	2	1
9	Yes, my neighbours eat snake meat	7	5
10	Yes, my neighbours kill snakes for medicinal purposes	14	9
11	Yes, all snakes around us should be killed	2	1
12	Yes, only venomous snakes around us should be killed	74	49
13	No, I do not consider snakes as friends of farmers	37	25
14	No, I do not think snakes need to be conserved	18	12
	c. Responses to ambivalent attitude test questions (*n* = 9) (i.e. ‘Yes’ responses to two or more questions that signify ambivalence)		
1	I like snakes in general/ I fear snakes in general	62	41
2	I fear snake/ I ignore snakes observed at premises of house and indoors	47	31
3	I worship snakes/ I kill or call others to kill snakes while I observed them in the crop field while working or on the path while walking	22	15
4	I worship snakes/ I prefer to kill whatever snakes I encounter anywhere	25	17
5	I like snakes in general/ I kill whatever snakes I encounter anywhere	9	6
6	I prefer to kill snakes/ all snakes should be conserved	7	5
7	All snakes should be killed/ only all venomous snakes should be killed/ all snakes should be conserved/ I think snakes should be conserved	52	35
8	I prefer to kill whatever snakes I encounter anywhere/ I consider snakes as farmers’ friends	15	10
9	I kill or call others to kill snakes I observe in the crop fields/ I consider snakes as farmers’ friends	14	9

*Abbreviation* and symbol: *N* number of respondents, *%* percent of respondents

**Fig. 4:**
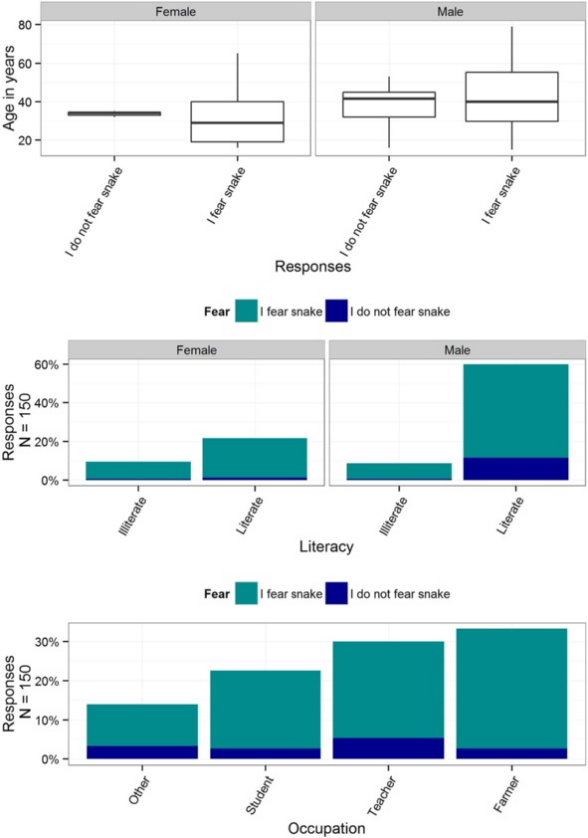
Fear of snakes among different demographic groups

Although 55 % of respondents (*n* = 82) were generally positive towards snakes (Table 6.a), 86 % (*n* = 129) feared snakes and 43 % (*n* = 64) were repulsed by snakes, primarily due to preconceptions about shape, size, and movements and related nightmares (44 %, *n* = 27, Table 7.b). Proportionately, males, literate persons, farmers, and teachers feared snakes more than their counterparts did (Fig. 4). We found a greater degree of negative attitudes towards snakes encountered indoors or in areas with increased human activity, such as homes, and agricultural fields. Thirty-eight percent (*n* = 57) of respondents would kill a snake if encountered, but this attitude varied by locality (Fig. 3). Only 1 % (*n* = 2) of respondents intended to kill all snake species encountered, but 49 % (*n* = 74) would kill all venomous snakes observed (Table 6.b). Approximately 6 % of respondents (*n* = 9) were negative towards snakes (median = 13, *p* = 0.037, Table 3.b), as indicated by answering two or more out of 14 questions on the negativity test (median = 2, *p* = <0.001, Table 4.b). Farmers, females, students, and illiterate people were the most negative towards snakes (Table 5.a.ii).

**Table 7:** Reasons for certain attitudes to snakes

SN	a. Major reasons of ‘I like snakes’ (frequency of respondents (f) = 64)	f	Percent
1	Snakes have attractive appearance and movement patterns (Attract), some snakes are non-poisonous (NP), prevent environmental pollution absorbing poison from environment (PEP), snakes do not bite until teasing (SUT)	22	34
2	Snake balances natural ecosystem and contribute to food-web (Ecosyst), snakes are farmer’s friends and important component of human beings (SFH), snakes are important component of biodiversity (Biod), snake venoms have medicinal value (Med), snakes are important for education (SIE), PEP	15	23
3	PEP	12	19
4	Pleasing God (“*Nag Devata*”), revering garland of Cobra worn by God Shiva as a God (God)	4	6
5	Biod, snakes attract tourist (AT)	3	5
6	Imitation (tradition) of worshipping snakes as a God by their predecessors or guardians (IP), ‘PEP’, God, snakes eat prey animals (rats, frogs, insects, etc.) (EP), Biod	3	5
7	PEP, Attract, NP	3	5
8	All snakes are not harmful (ASNH), snakes attract tourist (AT)	2	3
	b. Major reasons of ‘I dislike snakes’ (*f* = 62)		
1	I fear snakes’ shape, size, movement, dreams related to snakes, etc. (Fear)	27	44
2	Snake may bite any time, fear bite, it bites (Bite)	10	16
3	Snakes are poisonous (P)	9	15
4	Death after snakebite (DAB)	8	13
5	DAB, snakes are poisonous (P)	2	3
6	Snakes are dangerous animals (Danger)	2	3
7	Some snakes are venomous (SSV)	2	3
8	All snakes are dangerous (or harmful, venomous) (ASD), P	2	3
	c. Major reasons of ‘I worship snakes’ (*f* = 96) (note: respondents worshipping snakes without reasons (*f* = 30, 20 %)		
1	Imitated the practice of worshipping snakes by predecessors/parents (IP)	47	49
2	IP, God, prevention from witchcraft, witch and the Devil (PW), protection (Prot)	18	19
3	God	16	17
4	Prevention from snakebite or worshippig snakes might keep their trouble away (PB)	11	11
5	IP, wishing flourishment in the future (Wf)	4	4
	d. Major reasons of ‘I do not worship snakes’ (*f* = 11)		
1	No tradition of worshipping snake “*Nag*” in “*Nagpanchami*” (e.g., some Tharus) (NT)	7	64
2	Snakes do not lose natural potentiality of envenoming despite worshipping it (“Gadha dhoyara gai hudaina” i.e. black stone never turns white) (DNP)	2	18
3	It is duty of pandit (“Brahman” who is invited to worship serpent god) (DP)	2	18
	e. Major reasons of snake killing attitudes (*f* = 29)		
1	Kill venomous snakes only (KVO) because they are dangerous	9	31
2	Snake may bite any time, I fear from bite, it bites (Bite)	8	28
3	I fear from snakes’ shape, size, movement, dreams related to snakes, etc. (Fear)	7	24
4	Snakes are poisonous (P)	2	7
5	Snakes encountered might harm or disturb people (SEH)	2	7
6	Death after bite (DAB)	1	3
	f. Major reasons of ‘I do not kill any snakes’ (*f* = 81)		
1	I fear to kill/see snake, snake can chase (run) man (FK)	38	47
2	Neglecting encountered snakes without reasons (Ignore)	17	21
3	All snakes are not harmful (ASNH)	14	17
4	Snakes are symbol or representative of God (God)	4	5
5	Snakes do not bite until teasing them (SUT)	4	5
6	Snake does not attack (I do not kill snake until it attacks) (DA)	2	2
7	Snakes balance natural ecosystem and contribute to food-webs (Ecosyst)	2	2
	g. Major reasons of regarding snakes as friends of farmers (*f* = 60)		
1	Eats prey animals (e.g., rodents, insects, etc.) (EP)	50	83
2	EP, prevent environmental pollution absorbing poisonous gases (PEP)	6	10
3	EP, Snakes balance natural ecosystem and contribute to food-webs (Ecosyst)	4	7

Note: Respondents’ responses for why questions are grouped, coded and quantified in this table

Greater than 9 % of respondents (*n* = 14) were ambivalent towards snakes (median = 22, *p* = 0.04, Table 3.c) based on answering >1 of 9 questions on the ambivalence test (median = 2, *p* = <0.001, Table 4.c). In particular, males were more ambivalent towards snakes than females (Table 5.a.iii). Despite no difference in ambivalence towards snakes between teachers and farmers and teachers and students (Table 5.a.iii), teachers had the highest scores for ambivalence (median = 2, *p* = <0.001, Table 4.c).

### Knowledge

**Fig. 5:**
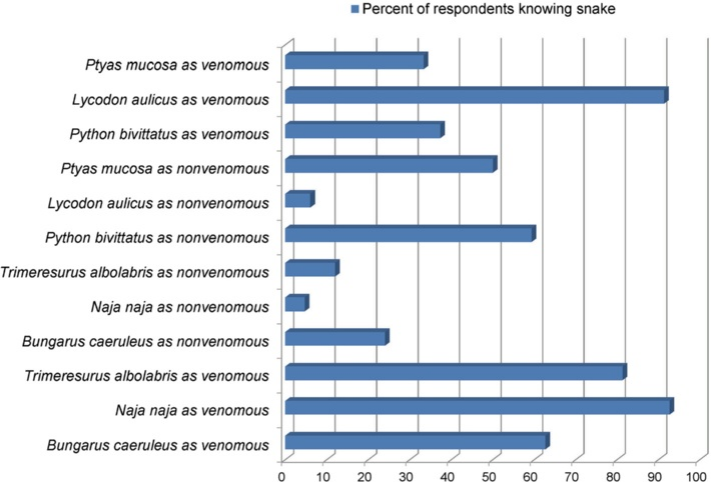
Knowing individual snakes by Chitwan National Park buffer zone people

Although inhabitants of CNPBZ were able to identify venomous (Table 8.a) more often than non-venomous snake species (Table 9.a), there remained substantial confusion in correct identification of snakes in general (Fig. 5, Table 8.b, 9.b, Table 10.b,d). Specifically, inhabitants correctly identified approximately 63 % (approximately 10 of 16 species) of venomous (median = 11, *p* = <0.001, Table 8.a) species, but only approximately 25 % (>3/12) of non-venomous snake species (median = 3, *p* = 0.001, Table 9.a). Inhabitants misidentified >50 % (>6/12) of non-venomous species as venomous (median = 7, *p* = <0.001, Table 8.b) and 19 % (>3/16) of venomous species to be non-venomous (median = 4, *p* = <0.001, Table 9.b). Only one (0.67 %) respondent thought all snakes were deadly venomous, and 5 % (*n* = 7) could not identify any non-venomous snakes.

**Table 8:** Familiarity of Chitwan National Park buffer zone people with native venomous snakes

Demographics		a. Correct scores for knowing venomous snakes (CSV, *n* = 16); null hypothesis (H0): population median scores (M) = hypothesized median scores (M0 = 10); alternative hypothesis (Ha): M > M0)			b. Incorrect scores for claiming non-venomous (ISV, *n* = 12) snakes to be venomous; null hypothesis (H0): population median scores (M) = hypothesized median scores (M0 = 6), alternative hypothesis (Ha): M > M0)		
		Median, range	W (CSV)	*p*-value	Median, range	W (ISV)	*p*-value
All respondents		11,4–16	6237.5	<0.001	7,1–12	5756.5	<0.001
Age (years)	15–24	11,7–15	612.5	<0.001	7,2–12	449.5	0.013
	25–34	10,4–16	86	0.181	5,2–11	121	0.431
	35–44	10.5,4–16	422.5	0.142	7,2–11	386.5	0.011
	45–54	12,6–14	136.5	0.048	7,1–10	115	0.214
	55–64	12,4–15	95	0.084	7,1–10	80	0.132
	65+above	11.5	22.5	0.285	9,3–11	30.5	0.045
Gender	Male	11,4–16	2567.5	0.070	6,1–11	2134.5	0.174
	Female	12,4–16	754.5	<0.001	8,3–12	843.5	<0.001
Occupation	Farmer	12,6–16	878.5	<0.001	8,1–11	815.5	<0.001
	Teacher	9,4–16	310.5	0.812	6,2–11	370.5	0.611
	Student	7,7–15	349	0.008	6,2–11	231.5	0.259
	Other^a^	12, 7–14	172	0.001	8,1–12	143	0.027
Educational status	Illiterate	12,6–16	256	0.006	9,3–11	369	<0.001
	Literate	11,4–16	3667.5	0.004	6,1–12	3144	0.062
	Class 10	12,4–15	301.5	0.012	7,1–12	245	0.039
	Class 11–12	11,7–16	253.5	0.059	5,2–11	169.5	0.687
	Master’s degree	10,4–16	98.5	0.730	6.5,2–11	157.5	0.280
	Bachelor’s degree	10.5,7–15	137	0.117	6.5,3–10	106.5	0.177
	Literate informally^b^	12,7–14	42	0.074	7,1–11	28	0.276

Symbols and *abbreviation*: ^a^hotel owner, miller, fisherman, boat-man, mason, labourer, housewife, nature guide; ^b^respondents able to read and write by informal education and never attained school; *n* number of snake species displayed, *W* value of one-tailed one-sample Wilcoxon signed rank test

**Table 9:** Familiarity of Chitwan National Park buffer zone people with native non-venomous snakes

Demographics		a. Correct scores for knowing non-venomous snakes (CSN, *n* = 12); null hypothesis (H0): median scores (M) = hypothesized median scores (M0 = 3), alternative hypothesis (Ha): M > M0			b. Incorrect scores of claiming venomous snakes (*n* = 16) to be non-venomous (ISN); null hypothesis (H0): population median scores (M) = hypothesized median scores (M0 = 3); alternative hypothesis (Ha): M > M0		
		Median, range	W (CSN)	*p*-value	Median, range	W (ISN)	*p*-value
All respondents		3,0–11	5303.5	0.001	4,0–11	5354	0.001
Age	15–24	2,1–7	172	0.998	4,1–9	580	0.001
	25–34	3.5,0–10	132	0.022	4,0–9	121	0.018
	35–44	2.5,0–10	314	0.510	3,0–10	318.5	0.038
	45–54	4,1–11	130	0.006	4,2–8	128	0.001
	55–64	4,1–9	76.5	0.177	3,1–11	52.5	0.151
	65+above	1.5,0–5	8	0.932	2,1–3	0	0.994
Gender	Male	4,0–11	3148	<0.001	4,0 –11	3045	<0.001
	Female	2,0–7	172	0.998	3,0–9	284.5	0.698
Occupation	Farmer	2,0–7	230	0.996	2.5,0–7	235.5	0.964
	Teacher	5,1–10	773	<0.001	5,0–11	679	<0.001
	Student	4,1–9	300	<0.001	4,1–9	469	<0.001
	Other^a^	2,0–11	80	0.604	3,2–8	62	0.036
Educational status	Illiterate	2,0–5	26	1.000	2,0–6	78.5	0.946
	Literate	4,0–11	4071.5	<0.001	4,0–11	3817.5	<0.001
	Class 10	3,0–9	170	0.424	3,1–11	131	0.296
	Class 11–12	4,1–9	250.5	<0.001	4,0–8	344.5	0.001
	Master’s degree	5,1–10	237	0.006	5,0–10	449.5	<0.001
	Bachelor’s degree	5,1–7	188	0.001	4,1–9	159.5	0.005
	Literate informally^b^	4,1–11	35	0.234	3,2–5	55	0.003

Symbols and *abbreviation*: ^a^hotel owner, miller, fisherman, boat-man, mason, labourer, housewife, nature guide; ^b^respondents able to read and write by informal education and never attained school; *n* number of snake species displayed, *W* value of one-tailed one-sample Wilcoxon signed rank test

**Table 10:** Familiarity of Chitwan National Park buffer zone people with native snakes, their knowledge about snake conservation and prevention of their bites

Group of people	a. Knowing VS		b. Claim of NVSasVS		c. Knowing NVS		d. Claim of VSasNVS		e. Knowing English name		f. Knowing local name		g. Snake conservation		h. Snakebite prevention	
	W	*p*-value	W	*p*-value	W	*p*-value	W	*p*-value	W	p-value	W	*p*-value	W	*p*-value	W	*p*-value
Younger and older^a^ (two tailed)	1214.5	0.994	1200	0.914	428.5	0.877	1199.5	0.911	1284.5	0.035	1107	0.450	1461	0.076	1533	0.026
Older^a^ > younger (one-tailed)	x	x	x	x	x	x	x	x	1831.5	0.018	x	x	x	x	x	x
Younger > older^a^ (one-tailed)	x	x	x	x	x	x	x	x	x	x	x	x	x	x	1533	0.013
Male and female (two tailed)	1620	0.001	1568	<0.001	3679	<0.001	3343.5	<0.001	1152	1	2358	0.716	3171.5	0.002	2861	0.091
Female > male (one-tailed)	3276	<0.001	3328	<0.001	x	x	x	x	x	x	x	x	x	x	x	x
Male > female (one-tailed)	x	x	x	x	3679	<0.001	3343.5	<0.001	x	x	x	x	3171.5	0.001	x	x
Farmer and student (two tailed)	1022.5	0.114	1145	0.007	328	<0.001	412	<0.001	720	0.014	1086.5	0.030	346	<0.001	324	<0.001
Farmer > student (one-tailed)	x	x	1145	0.003	x	x	x	x	x	x	1086.5	0.015	x	x	x	x
Student > farmer (one-tailed)	x	x	x	x	1372	<0.001	1288	<0.001	980	0.007	x	x	1354	<0.001	1376	<0.001
Farmer and teacher (two tailed)	1543.5	0.002	1551.5	0.001	483.5	<0.001	457	<0.001	649.5	<0.001	1632.5	<0.001	380	<0.001	483.5	<0.001
Farmer > teacher (one-tailed)	1543.5	0.001	1551.5	0.001	x	x	x	x	x	x	1632.5	<0.001	x	x	x	x
Teacher > farmer (one-tailed)	x	x	x	x	1766.5	<0.001	1793	<0.001	1600.5	<0.001	x	x	1870	<0.001	1766.5	<0.001
Teacher and student (two tailed)	1000.5	0.019	836	0.482	696.5	0.496	641	0.217	520	0.004	912.5	0.143	787	0.819	673	0.353
Students > teacher (one-tailed)	1000.5	0.009	x	x	x	x	x	x	x	x	x	x	x	x	x	x
Teacher > student (one-tailed)	x	x	x	x	x	x	x	x	1010	0.002	x	x	x	x	x	x
Literate and illiterate (two tailed)	2039.5	0.035	2420	<0.001	660	<0.001	887.5	<0.001	1118	0.003	1883.5	0.185	610.5	<0.001	750.5	<0.001
Illiterate > literate (one-tailed)	2039.5	0.017	2420	<0.001	x	x	x	x	x	x	x	x	x	x	x	x
Literate > illiterate (one-tailed)	x	x	x	x	2580	<0.001	2352.5	<0.001	2052	0.001	x	x	2629.5	<0.001	2489.5	<0.001

Symbols and *abbreviation*: ^a^15–34 years old people are considered younger and 45–64 years as older people; *VS* venomous snakes (including mildly and highly venomous ones), *NVS* non-venomous snakes, *null hypothesis (H0): population median score (M)* hypothesised population score (M0 = 0) (i.e. H0: M = M0), *alternative hypothesis.1 (Ha.1)* population median score (M) ≠ hypothesised population score (M0), *alternative hypothesis.2 (Ha.2)* population median score (M) > hypothesised population score (M0), *W* value for two-tailed and one-tailed unpaired Wilcoxon rank sum test

Females correctly identified more venomous snakes than males (median = 12, *p* = <0.001, Table 8.a), but males identified more non-venomous snakes than females (Table 9.a). Farmers identified more venomous snakes (median = 12, *p* = <0.001) than teachers (median = 9, *p* = 0.812, Table 8.a, Table 10.a). Students had the best aptitude for identifying venomous snakes among all occupational groups (Table 10.a). Illiterate people correctly identified more venomous snakes, but literate respondents correctly identified more non-venomous snakes (Table 10.a,c).

**Fig. 6:**
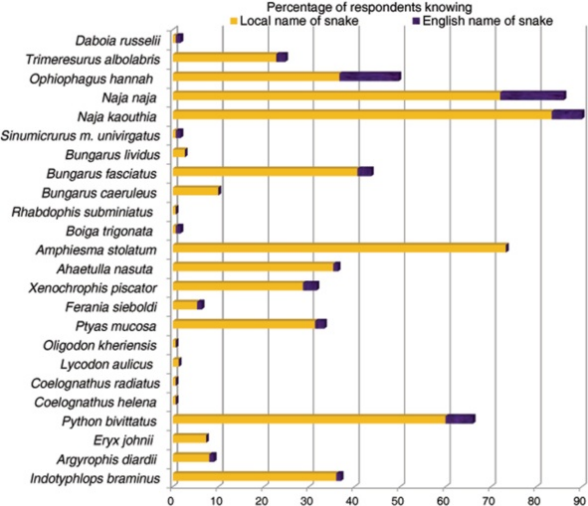
Knowing snakes by English and local name by Chitwan National Park buffer zone people

Respondents incorrectly identified Common Kraits (*Bungarus caeruleus*, 24 %), Common Cobras (*Naja naja,* 5 %) and Green Pit Vipers (*Trimeresurus albolabris*, 12 %) as non-venomous species. Ninety-one percent of respondents wrongly thought that Common Wolf Snakes (*Lycodon aulicus*) were venomous, with 66 % identifying the species as kraits (*Bungarus* spp.); 33 % thought Rat Snakes (*Ptyas mucosa*) were venomous and 2 % identified them as Cobras (*Naja* spp.); 59 % of respondents correctly identified Pythons (*Python bivittatus*) as non-venomous, and 37 % thought they were venomous (Fig. 5). Almost no respondents were able to identify snakes by their scientific and English names, and they were only slightly familiar with local names (Fig. 6). Older people, students, teachers, and literate people knew the English names of snakes more often than other groups (Table 10.e, Table 11.a). Conversely, farmers knew local names of snakes more often (Table 10.f, Table 11.b). No respondents knew the name of the Saw-scaled Viper (*Echis carinatus*) and only 1 % of respondents knew the local and English names for *D. russelii*. In total, 83 % of respondents knew the local name for the Monocellate Cobra (*Naja kaouthia*), 72 % for the Common Cobra (*N. naja*), 23 % for the Green Pit Viper (*T. albolabris*), 73 % for the Striped Keelback (*Amphiesma stolatum*), and 60 % for the Python (*P. bivittatus*) (Fig. 6).

**Table 11:** Familiarity of Chitwan National Park buffer zone people with names of native snakes (*n* = 28)

Demographics		a. Knowing snakes by their English name (KEN); null hypothesis (H0): population median scores (M) = hypothesized median scores (M0 = 1); alternative hypothesis (Ha): M > M0			b. Knowing snakes by their local name (KLN); null hypothesis (H0): population median scores (M) = hypothesized median scores (M0 = 5), alternative hypothesis (Ha): M > M0		
		Median, range	W (KEN)	*p*-value	Median, range	W (KLN)	*p*-value
All respondents		0,0–12	2888	1	6,0–14	5625	0.004
Age (years)	15–24	0,0–2	82	1	5,1–11	414.5	0.171
	25–34	0,0–7	91.5	0.891	5,0–11	91.5	0.405
	35–44	0,0–8	236	0.990	6,2–11	508.5	0.009
	45–54	0,0–12	93	0.376	7,1–14	150	0.003
	55–64	0,0–4	42.5	0.965	5,1–7	39	0.892
	65+ above	0	0	0	5,3–7	16	0.641
Gender	Male	0,0–12	1780.5	0.985	7,5–11	2582	0.026
	Female	0,0–3	0	0	6,0–11	597.5	0.033
Occupation	Farmer	0,0–3	0	0	6,0–11	824	<0.001
	Teacher	0,0–6	558.5	0.142	4,1–9	253.5	0.974
	Student	0,0–2	66	1	5,1–11	239.5	0.319
	Other^a^	0,0–12	0	0	7,1–14	203	0.001
Educational status	Illiterate	0	0	0	6,0–11	257.5	0.005
	Literate	0,0–12	2243	0.999	5,1–14	3277.5	0.056
	Up to class 10	0,0–12	0	0	6,1–14	298.5	0.001
	Class 11 to 12	0,0–2	56	1	4,1–9	174	0.751
	Master’s degree	1,0–6	240.5	0.046	4,1–9	90.5	0.930
	Bachelor’s degree	0,0–3	83	0.939	5,1–11	116	0.203
	Literate informally^b^	0,0–8	0	0	7.5,3–10	42	0.012

Symbols and *abbreviation*: ^a^hotel owner, miller, fisherman, boat-man, mason labourer, housewife, nature guide; ^b^respondents able to read and write by informal education but never attained school; *n* number of snake species displayed, *W* value of one-tailed one-sample Wilcoxon signed rank test. I zeroed W and *p* values for confidence interval (CI) below 95%

A total of 81 % (*n* = 122) of CNPBZ inhabitants considered there to be a need for snake conservation (Table 7.a), but respondents below 35 years of age, teachers, students, and literate people only gave one reason to justify the need for snake conservation (Table 12.a). Although 85 % (*n* = 127) of respondents replied that they were aware of preventive measures for snakebite, their reasoning was poor. Respondents aged 15–24 years, teachers, students, and literate people mentioned slightly more than two appropriate preventive measures (Table 12.b), although their score for “yes” responses was higher. Snake conservation knowledge was greater among males (Table 10.g), and knowledge about snakebite prevention was greater among younger respondents (Table 10.h).

**Table 12:** Knowledge of Chitwan National Park buffer zone people about the need of snake conservation and preventive measures against snakebites

Demographics		a. Scores for logics explaining the need of snake conservation (cons., *n* = 5); null hypothesis (H0): population median scores (M) = hypothesized median scores (M0 = 1), alternative hypothesis (Ha): M > M0			b. Scores for appropriate preventive measures mentioned (prev., *n* = 10); null hypothesis (H0): population median scores (M) = hypothesized median scores (M0 = 2), alternative hypothesis (Ha): M > M0		
		Median, range	W (cons.)	*p*-value	Median, range	W (prev.)	*p*-value
All respondents		1,0–4	2064	0.626	2,0–9	3678.5	0.210
Age (years)	15–24	1,0–4	251.5	0.055	2.5,0–7	398	0.005
	25–34	1.5,0–4	103.5	0.028	2.5,0–6	107.5	0.170
	35–44	1,0–3	66.5	0.904	2,0–9	225	0.568
	45–54	1,0–3	24	0.909	1,0–7	51	0.824
	55–64	1,0–4	27.5	0.720	2,0–4	49	0.602
	65+above	0,0–2	3.5	0.960	0,0–2	0	0
Gender	Male	1,0–4	1042	0.099	2,0–9	1935	0.062
	Female	0,0–3	158	0.992	2,0–4	264	0.872
Occupation	Farmer	0,0–2	80	1	1,0–6	169	1
	Teacher	1,0–4	199	0.001	2,0–9	338.5	0.004
	Student	1,0–4	162	0.013	3,0–7	317.5	0.001
	Other^a^	1,0–2	0	0	2,0–7	78	0.481
Education status	Illiterate	0,0–2	0	0	0,0–6	55	0.999
	Literate	1,0–4	1423	0.028	2,0–9	2675	0.002
	Class 10	0,0–4	0	0	1,0–5	144.5	0.797
	Class 11 or 12	1,0–4	150.5	0.037	3,1–7	295	<0.001
	Master’s degree	1,0–4	69	0.008	2,0–9	89.5	0.009
	Bachelor’s degree	1,0–4	57	0.015	2,0–6	77.5	0.317
	Literate informally^b^	1,0–2	0	0	1,0–7	14.5	0.717

Symbols and *abbreviation*: ^a^hotel owner, miller, fisherman, boat-man, mason, labourer, housewife, nature guide; ^b^respondents able to read and write by informal education and never attained school; *n* number of logical statements to support their “Yes” reply to the need of snake conservation and preventive measures of snake bite, *W* value for one-tailed one-sample Wilcoxon signed rank test. We zeroed W and *p* values for confidence interval below 95%

### Awareness

Awareness of recommended pre-hospital care of snakebite was below 50 % on average. We found that >48 % of respondents aware of proper snakebite care (*n* = 72, *p* = 0.045, Table 13) based on rejecting traditional beliefs and medical care of doubtful use, and accepting modern measures of pre-hospital care of snakebite (median = 19, *p* = 0.001, Table 14.a). In contrast, >21 % (*n* = >32) of respondents were unaware of recommended pre-hospital care of snakebite (*p* = 0.033, Table 13) as indicated by accepting traditional beliefs and medical help of doubtful use, and rejecting modern measures of pre-hospital care (median = 9, *p* = <0.001, Table 14.b). More than 11 % (*n* = >17) respondents were unfamiliar (*p* = 0.034) with awareness test questions (i.e., they did not know the correct answer for >3 awareness test questions; median = 3.5, *p* = <0.001, Table 14. Note 1.).

**Table 13:** Chitwan National Park buffer zone people responding to misbeliefs on snakes, traditional and modern care of snakebites

		General responses	Median, range	W (resp)	*p*-value	Median ^a^	Median %	LA^b^
a. All respondents’ responses (*n* = 33, see Table 15)		Aware (not believing on misbelief but believing on recommended care), H0 (A): M = M0 (72), Ha (A): M > M0 (72)	82,4–142	335	0.045	72	48	MA
		Unaware (believing on misbelief but not believing on recommended care), H0 (UA): M = M0 (32), Ha (UA): M > M0 (32)	40,2–141	362.5	0.033	32	21	
		Unknown to both traditional and modern information, H0 (Uk): M = M0 (17), Ha (Uk): M > M0 (17)	24,1–61	362	0.034	17	11	
		Not answered to both traditional and modern information (i.e. item nonresponses), H0 (NA): M = M0 (1), Ha (NA): M > M0 (1)	2,0–11	417.5	0.002	1	1	
b. Categorical responses	*n* = 28, see Table 15.a–c	Aware (not believing misbelief on snakes and snakebite care), H0 (A28): M = M0 (67), Ha (A28): M > M0 (67)	75,4–142	280.5	0.040	67	45	MA
		Unaware (believing on misbelief on snakes and snakebite care), H0 (UA28): M = M0 (35), Ha (UA28): M > M0 (35)	43,2–141	278	0.045	35	23	
	*n* = 13, see Table 15.a	Aware (not believing misbelief on snakes), H0 (A13): M = M0 (62), Ha (A13): M > M0 (62)	72,39–142	62.5	0.036	62	41	MA
		Unaware (believing misbelief on snakes), H0 (UA13): M = M0 (28), Ha (UA13): M > M0 (28);	43,2–82	71.5	0.037	28	19	
	*n* = 15, see Table 15. b,c	Aware (not believing on traditional and doubtful pre–hospital care of snakebite), H0 (A15): M = M0 (60), Ha (A15): M > M0 (60)	76,4–128	90.5	0.044	60	40	MA
		Unaware (believing on traditional and doubtful pre–hospital care of snakebite), H0 (UA15): M = M0 (33), Ha (UA15): M > M0 (33)	43,18–141	91.5	0.039	33	22	
	pre–hospital care (*n* = 5, see Table 15.d)	Aware (believing the recommended pre–hospital care of snakebite), H0 (A5): M = M0 (76), Ha (A5): M > M0 (76)	125,77–142	15	0.031	76	51	A
		Unaware (not believing on recommended pre–hospital care of snakebite), H0 (UA5): M = M0 (1), Ha (UA5): M > M0 (1)	22,2–47	15	0.031	1	1	

Symbols and *abbreviations*: ^a^median significantly greater than (after hypothesis test), ^b^level of awareness, *%* percent, *W(resp)* one-tailed one-sampled Wilcoxon value of respondents who responded particular belief on snakes and/or care of snakebites, *H0* null hypothesis, *Ha* alternative hypothesis, *M* population median, *M0* hypothesized median (parenthesis contains figure of hypothesized median), *UA* unaware (0–24 %), *MA* slightly aware (25–49 %), *A* aware (50–74 %), *Uk* Unknown, *NA* Not answered, *resp* respondents

**Table 14:** Awareness of Chitwan National Park buffer zone people concerning belief on snakes and snakebite care

Demographics		a. Scores for awareness (rejecting traditional belief and medical help of doubtful use and accepting modern measures of pre-hospital care, *n* = 33); null hypothesis (H0): population median scores (M) = hypothesized median scores (M0 = 16), alternative hypothesis (Ha): M > M0(16)			b. Scores for unawareness (accepting traditional belief and medical help of doubtful use and rejecting modern measures of pre-hospital care, *n* = 33); null hypothesis (H0): population median scores (M) = hypothesized median scores (M0 = 8), alternative hypothesis (Ha): M > M0(8)		
		Median, range	W (Aware)	*p*-value	Median, range	W (Unaware)	*p*-value
All respondents		19,3–31	6564.5	0.001	9,0–24	6628	<0.001
Age (years)	15–24	16,7–28	433.5	0.182	11,2–18	628	<0.001
	25–34	20.5,3–31	178	0.049	8,2–23	98.5	0.452
	35–44	19,4–30	394.5	0.260	8.5,2–12	470.5	0.132
	45–54	21,6–29	193.5	0.004	8,2–14	94	0.667
	55–64	21,9–27	111	0.014	10,3–24	114.5	0.037
	65 and above	9,3–25	8	0.930	9,0–17	19	0.223
Gender	Male	20,3–31	3769.5	<0.001	8,0–24	2698.5	0.039
	Female	13.5,3–27	335.5	0.952	10,2–23	855.5	<0.001
Occupation	Farmer	12,3–28	435.5	0.975	9.5,0–24	873.5	0.005
	Teacher	22,14–30	898	<0.001	8,2–17	382	0.650
	Student	16,7–28	271	0.329	11,2–18	426.5	<0.001
	Other^a^	21,5–31	142.5	0.029	9,2–22	126.5	0.216
Educational status	Illiterate	10,3–28	75	0.997	10,0–23	227.5	0.005
	Literate	20,5–31	4954	<0.001	9,2–24	3982	0.005
	Up to class 10	22,5–31	388	0.001	7,2–21	165.5	0.918
	Class 11 or 12	17,7–29	734	0.068	12,3–18	376.5	<0.001
	Master’s degree	22,16–30	325	<0.001	7,3–17	123	0.682
	Bachelor’s degree	21,13–28	220	<0.001	9,2–15	135.5	0.129
	Literate informally^b^	12.5,7–26	25	0.620	12.5,4–24	48.5	0.018

Symbols and *abbreviations*: ^a^hotel owner, miller, fisherman, boat–man, mason, labourer, housewife, nature guide; ^b^respondents able to read and write by informal education but never attained school, *n* total number of awareness test questions, *W* value of one-tailed one-sample Wilcoxon signed rank test. *Note:* 1. Scores for unknown (to traditional belief, medical help of doubtful use and modern measures of pre-hospital care (median = 3.5, range = 0–30, *p* = <0.001); 2. Scores for not answered (any questions regarding traditional belief, medical help of doubtful use, and modern measures of pre-hospital care (item non–responses) (median = 0, range = 0–16, *p* = <0.001)

Respondents were highly aware of particular practices of snakebite care and belief. Out of 33 awareness test questions (Table 15), 95 % of respondents were aware of the need to visit a treatment center equipped with antivenom (95 %) to treat snakebite from venomous species (88 %, *n* = 132), and they also knew where the nearest snakebite treatment center was located (83 %, *n* = 125). Similarly, 95 % of respondents rejected the belief that all snakes are venomous and 85 % refused to seek treatment from traditional healers (Table 15). More than half of respondents accepted widely recommended first aid measures for venomous snakebite. However, a total of 61 % of respondents would apply PIB and 51 % would apply LCPI (Table 15). Overall, only >51 % of respondents (*n* = >76) agreed to apply suggested first aid measures (median = 125, *p* = 0.031, Table 13.b). The level of awareness of proper snakebite treatment was greater among males than females, among student and teachers than farmers, among teachers than students, and among literate than illiterate people (Table 5.b.i).

### Ethno-ophiology

Ninety-nine percent of respondents replied that they made no use of snakes and snake products, and only 2/150 (1 %) respondents consumed *Python* meat. Five percent of respondents knew neighbors from native ethnic groups (i.e., Tharu, Mushahar, Kusunda, and Newar) that consumed snake meat, and 9 % (*n* = 14) of respondents knew about the killing of *Pythons* for gallbladder and fat, and Cobras for fat and intestines used to purportedly cure backache, burn or other infected wounds, hemorhoids, mastitis, and rheumatism (Table 6).

## Discussion

Ignorance of the need for snake conservation, extreme disgust and fear of snakes (Fig. 4), a strong desire to kill snakes, confusion in differentiating venomous from non-venomous species (Fig. 5), a willingness to exterminate all venomous snakes by nearly 50 % of respondents, and susceptible positivity to snakes appear to be challenges to snake conservation in the lowlands of Nepal. Killing and harassing snakes is common in CNPBZ [51] and elsewhere in Nepal [36]. General belief that most snakes are venomous and the desire to kill snakes is also known to occur in neighboring areas, such as Sikkim state in India [52]. Similar challenges were apparent in Brazil [53], Kenya [54], and Australia [14]. Indeed, wanton killing of snakes appears to be a worldwide phenomenon, which likely amplifies wide-ranging declines in snake population [13, 55], and only serves to heighten the importance of snake conservation. Continued killing of snakes may negatively impact snake population dynamics, potentially resulting in a trophic cascade that leads to deterioration of ecosystems, which may ultimately impact human health. Extinction of species due to factors, such as climate change [56] may add to the global biodiversity crisis [57]. Therefore, authorities should consider human-snake conflicts as an important driver of snake population declines.

Killing of snakes appears to be the result of extreme negativity that originates due to fear. A lack of awareness about essential ecological services that snakes provide facilitates the killing of snakes. Although 95 % of the respondents in the current study were aware that all snakes are not venomous, more than two-thirds of respondents feared snakes. Furthermore, a lack of awareness of the need for snake conservation, and extreme fear of snakes (especially among farmers) has apparently led to large-scale killing of snakes in Nepal [58]. Increased tolerance of snakes in areas with less human activity further suggests that fear of snakes leads to human-caused mortality of snakes in Nepal. The fear of snakes may have originated since time immemorial [59], and was introduced into the scientific community by Linnaeus [60]. Irrational belief in snake mythologies propagates fear and negativity towards snakes [61, 62]. Revulsion, accompanied by a lack of awareness about snakebite prevention, and inappropriate care of snakebites due to superstition likely induce fear of snakes in humans, which is compounded by the possible fear of snakes that was evolutionarily ingrained in the human brain [63, 64]. Further, poor transportation, ill-equipped and inaccessible snakebite care facilities, and greater snakebite mortality as a consequence [65, 66]), likely intensifies fear of snakes. Moreover, a famous Nepali proverb “*Bish nabhyako sarpa ra ekh nabhyako manis hudaina* (i.e., “there are no snakes without venom and no humans without jealousy”) likely fortifies fear, leading to increased negativity towards snakes. And finally, a lack of understanding of ecological services provided by snakes appears to cultivate negativity, which predisposes people to kill snakes.

Similar to teachers in Kenyan communities [54] and students in Brazilian communities [53], a fear of snakes is common in Nepal. Coupling fear and negativity with a poor aptitude for distinguishing venomous and nonvenomous snakes likely results in wanton killing of all snakes, including non-venomous species, in order to feel safe. In Nepal, killing snakes is common, even in and around biodiversity conservation hotspots [36, 51]. Similar ruthless killing of snakes reported from other parts of Nepal [67], and intentional killing of snakes encountered elsewhere in our study, suggests a lack of education about snake ecology and effective practices of snakebite prevention and treatment.

Due to the relatively high rate of respondents that believed pythons are venomous places this species at risk from human-caused mortality. Less familiarity of snakes by teachers and students suggests the need for intensive educational efforts designed to minimize wanton killing of snakes. The fact that teachers and students, who are the backbone of the community, fail to recognize the difference between venomous and non-venomous snakes may impact snake conservation and public health in Nepal and elsewhere with similar circumstances.

In contrast to China, Vietnam, Brazil [4, 68, 69], and other parts of Nepal [51, 70], the use of snakes for food and medicinal products does not appear to be a significant threat to snake conservation in the Chitwan Valley. Killing snakes for human use was rare in Nepal compared to past reports [67]. Similar to some areas in India [71], only people from sparsely populated ethnic communities used pythons and cobras as food and/or medicine. The majority of Tharu respondents in our study denied the eating of snake meat, although Zug and Mitchell mentioned that the Tharus of Chitwan were known to consume snakes [51]. However, there is need for intensive research on the ethno-medicinal use of snake products nationwide.

Humans have long been suffering from the consequences of our attitudes towards snakes [61]. Fear, bias, negativity, disregard, and superstition are behavioral risk factors for snakebite in Nepal and elsewhere with similar geosocioeconomic and cultural circumstances. Ignorant, negative, and fearful people who tend to encourage the killing of snakes put themselves at the risk of envenomation. Pronounced confusion in differentiating venomous and non-venomous snakes, even among teachers and students in Nepal, teachers in Kenya [54] and medical service providers in Sri Lanka [72] disclose the need for more effective education in southern Nepal and elsewhere with similar circumstances.

Like in this study, the inability to correctly identify snakes, or the illogical claim of being able to identify snakes [66], also occurs in developed countries [73]. People commonly identify non-venomous snakes as venomous [74]. This increases the risk of and vulnerability of rural inhabitants to snakebite envenomation. Therefore, finding out which of the snakes in residential or visiting area are venomous and which are not is essential. Accurate identification of snake species not only enhances snakebite prevention, may minimize the chance of multiple bites to a victim or a bite to the first aid provider. Correctly identifying snakes can also minimize snakebites for people who attempt to kill the snake. If snakes are mishandled, envenoming by recently killed, decapitated or inadequately killed [75], preserved [76], and even frozen specimens [77] is possible. A lack of identification skills and knowledge about preventive measures against snakebite undoubtedly places inhabitants of agrarian lowlands in Nepal at increased risk of snakebite. Our findings that a large majority of people who claimed to be familiar with recommended snakebite preventive measures actually had very little idea of how to prevent snakebites. Although farmers and illiterate people may be expected to be at higher risk of snakebite, the fact that people with higher education were also at greater risk indicates that ignorance about snakes and snakebite are widespread among all sectors of the community, leading us to conclude that education about snakes and snakebite prevention at schools, universities [46] and within the community is inadequate.

The relatively greater degree of negativity towards snakes among farmers, females, students, and illiterate people (Table 5.a.ii) may be associated with a lack of knowledge about the role that snakes play in ecosystems, the risk of snakebite, and proper preventative measures. The high degree of ambivalence towards snakes among teachers is especially troubling, because it indicates that educators are likely not utilizing factual information on snakes and snakebite issues to develop effective educational approaches.

On a positive note, 85 % of respondents rejected traditional healers as a legitimate alternative treatment for snakebite, which is supported by a decreasing trend of dependency on traditional healers for snakebite treatment in the Chitwan Valley [66, 78, 79]. Therefore, we conclude that traditional healing is not a challenge to snakebite management in this part of Nepal. However, the situation is different in other countries. For example, 86 % of snakebite victims in Bangladesh [80], 75 % in Pakistan [81], and 61 % in India [82] still visit traditional healers. Belief in traditional snakebite treatment methods may be a challenge to snakebite management in other regions of Nepal. Although CNPBZ inhabitants scored slightly above average scores for their choice of recommended first aid measures (e.g., PIB, LCPI), likely due to recent training workshops and related books, poster, and pamphlet distribution in 2007, 2008, 2009, and 2011 [79], a recent hospital-based snakebite report from southwestern Nepal [83] and central Nepal [84] reported the common use of inappropriate snakebite first aid. Inadequate education related to the pre-hospital care of snakebite [46] likely influences chosen practices of pre-hospital care of snakebite. However, we suggest intensive research on the locally available (currently unreported) ethno-biological snakebite remedies because these home remedies may pacify snakebite victim, slow down pulse rate that slows down venom dissemination. This eventually keep patient less danger and prolong time to admit in snakebite treatment center before severe venom effect [74].

In evaluating weaknesses of our survey, we minimized sampling and non-sampling errors to the best of our ability by involving qualified interviewers, carrying out interviews primarily in isolation, and cross checking data entry to avoid measurement errors. We did not face any unit (respondent) non-responses (standard response rate = 1), but we noted item non-responses for certain questions (Table 6.b, 13.a, 14. Note 2, 15), likely due to lack of knowledge on the part of respondents. The item non-response for educational status was three. We provided non-monetary incentive of books about snakes and snakebites [45, 85] to each respondent (Fig. 2), which motivated them to participate in the survey and maximized the response rate. Further, we scheduled interviews at times when villagers were either less hectic or more involved in household chores to manage the already harvested crops, thereby maximizing the number of responses.

**Table 15:** Responses of Chitwan National Park buffer zone people to awareness test questions

SN	a. Traditional belief on snakes (those which are potentially cause snakebites are Italicized)	Responses (% percent, N number of respondents)						Level of awareness
		I believe (N)	I believe (%)	I don’t believe (N)	I don’t believe (%)	I don’t know (N)	Item non–responses (N)	
1	All snakes surrounding us are venomous	2	1	142	95	6	0	HA
2	Snakes can have rebirth	22	15	98	65	30	0	A
3	Snakes can hypnotize	15	10	96	64	39	0	A
4	View of snake on the way/journey bode good future	34	23	92	61	24	0	A
5	After bites, snakes go to tree-top to view victim’s funeral	7	5	91	61	52	0	A
6	Snakes eyes can photograph to take revenge	43	29	86	57	21	0	A
7	Kill partner of snake to avoid revenge of survived ones	59	39	72	48	19	0	MA
8	Snakes possess invaluable stone ‘Mani’	53	35	63	42	34	0	MA
9	Snakes can suckle milk from cows, goats, or sheep	57	38	62	41	30	1	MA
10	Some snakes guard the property of people	59	39	61	41	30	0	MA
11	Vine snakes bite only on eye or forehead	55	37	58	39	37	0	MA
12	There are two-mouthed snakes	40	27	49	33	61	0	MA
13	Snakes (e.g., cobras) can dance in tune of music	82	55	39	26	29	0	MA
	b. Traditional belief on pre-hospital care							
1	Visiting traditional healers	18	12	128	85	2	2	HA
2	Sucking wound	34	23	108	72	5	3	A
3	Applying other traditional concoction topically	25	17	90	60	31	4	A
4	Squeezing the wound	47	31	88	59	13	2	A
5	Ingesting other traditional concoction	32	21	85	57	27	6	A
6	Applying the cloaca of chickens	28	19	82	55	34	6	A
7	Ingesting chillies	45	30	82	55	19	4	A
8	Applying honey on the site of bite	20	13	76	51	49	5	A
9	Incising bite site	62	41	74	49	12	2	MA
10	Ingesting herbal medicine	40	27	74	49	31	5	MA
11	Applying herbal medicine topically	43	29	72	48	31	4	MA
12	Using snake stone	47	31	63	42	36	4	MA
13	Applying (tight) tourniquet	95	63	48	32	4	3	MA
	c. Seeking medical help of doubtful use							
1	Visiting medical person	133	89	5	3	1	11	UA
2	Visiting any hospital or healthcare centre	141	94	4	3	1	4	UA
	d. Recommended measures of pre-hospital care							
1	Visiting healthcare facilities supplied with antivenom	142	95	2	1	3	3	HA
2	Envenomation can be cured by antivenom	132	88	8	5	7	3	HA
3	Availability of nearby snakebite treatment centre	125	83	22	15	3	0	HA
4	Pressure immobilization bandaging (PIB)	92	61	40	27	12	6	A
5	Local compression pad immobilization (LCPI)	77	51	47	31	17	9	A

Awareness level: *UA* unaware (0–24 %), *MA* slightly aware (25–49 %), *A* aware (50–74 %), *HA* highly aware (75–100 %)

Photographs and captive specimens are deprived of additional ecological information that may help to a positive identification of the specimen [86, 87]. Due to crepuscular and nocturnal habits of some snakes, which cause about 25 % of snakebites at night [66], they may not be visible clearly. As photographs subtracts the ecological information associated with specimens [87], this may increase the chance for misclassifying the materials. Nonetheless, ecological circumstances and habitat associated with those snakes can, in fact, be recognized by respondents. Thus, there is a need for trekking with respondents in nature park or serpentarium to gather more reliable data.

## Conclusion

The cumulative effect of fear, antipathy, negativity, ignorance, and ambivalence to snakes among people represent potential threats to snake conservation. Apparent decline of local snake populations and extirpation of rare or endangered snake species in the lowlands of Nepal may occur if wanton killing of snakes is unchecked, which has multifarious and unforeseen negative impacts on biodiversity and human health. Therefore, potential factors responsible for large-scale killing of snakes should be considered when developing biodiversity conservation and public health strategies. Increasing knowledge and the awareness of people about snake and snakebite care and prevention through educational interventions, such as snake parks and snake museums, are cost effective ways of developing snake friendly attitudes of people. The ability to recognize venomous from non-venomous snakebites should be considered in future studies, as this ability can be crucial in the decision to of whether or not to seek immediate medical attention.

## Abbreviations

AKA: attitudes, knowledge and awareness of native snakes, snake conservation, care and prevention of snakebites
BZ: buffer zones
CNP: Chitwan National Park

## Footnotes

1 Occupation of respondents was defined based on the work that they carried out within the previous 12 months. Therefore, the occupation of respondents for those 12 months and some interference may be encountered if case-by-case life histories were taken into account.

## References

[1] Moura MR, Costa HC, São-Pedro VA, Fernandes VD, Feio RN (2010). The relationship between people and snakes in eastern Minas Gerais, southeastern Brazil. Biota Neotrop.

[2] Alves RRN (2012). Relationships between fauna and people and the role of ethnozoology in animal conservation. Ethnobiol Conserv..

[3] Miller H (1970). The cobra, India’s “good snake”. Natl Geogr..

[4] Somaweera R, Somaweera N (2010). Serpents in jars: the snake wine industry in Vietnam. JoTT.

[5] Mendonça LET, Vieira WLS, Alves RRN (2014). Caatinga ethnoherpetology: relationships between herpetofauna and people in a semiarid region. Amphibian and Reptile Conserv.

[6] Gordon GB (1905). The serpent motive in the ancient art of central America and Mexico. T Dept Archaeol, University of Pennsylvania.

[7] Hastings J, Selbie JA, Gray LH, Encyclopaedia of religion and ethics (1922). Serpent-worship. Encyclopaedia of religion and ethics.

[8] Sasaki K, Sasaki Y, Fox SF (2010). Endangered traditional beliefs in Japan: influences on snake conservation. Herpetol Conserv Biol.

[9] Alves RRN, Souto WMS (2015). Ethnozoology: a brief introduction. Ethnobiol Conserv.

[10] Alves RRN, Vieira WLS, Santana GG (2008). Reptiles used in traditional folk medicine: conservation implications. Biodivers Conserv.

[11] Alves RRN, Albuquerque UP (2012). Ethnobiology and conservation: why do we need a new journal?. Ethnobiol Conserv..

[12] Gibbons JW, Scott DE, Ryan TJ, Buhlmann KA, Tuberville TD, Metts BS, Greene JL, Mills T, Leiden Y, Poppy S (2000). The global decline of reptiles, déjà vu amphibians. Bioscience.

[13] Godley JS, Moler PE (2013). Population declines of Eastern Indigo Snakes (*Drymarchon couperi*) over three decades in the gulf Hammock Wildlife Management Area, Florida, USA. Herpetol Conserv Biol..

[14] Whitaker PB, Shine R (2000). Sources of mortality of large elapid snakes in an agricultural landscape. J Herpetol.

[15] Boehm M, Collen B, Baillie JEM, Bowles P, Chanson J, Cox N, et al. The conservation status of the world’s reptiles. Biol Conserv. 2013;157:372–85.

[16] Seigel RA, Mullin SJ, Mullin SJ, Seigel RA (2009). Snake conservation, present and future. Snakes, ecology and conservation.

[17] Carter NH, Riley SJ, Shortridge A, Shrestha BK, Liu J (2013). Spatial assessment of attitudes toward tigers in Nepal. J Human Environ.

[18] Mills LS, Soulé ME, Doak DF (1993). The keystone-species concept in ecology and conservation. Bioscience.

[19] Kotliar NB, Baker BW, Whicker AD, Plumb G (1999). A critical review of assumptions about the prairie dog as a keystone species. Environ Manag.

[20] Beaupre SJ, Douglas LE, Mullin SJ, Seigel RA (2009). Snakes as indicators and monitors of ecosystem properties. Snakes: ecology and conservation.

[21] Brown PR, Nyunt-Yee, Singleton GR, Kenney AJ, Nyo-Me-Htwe, Myo-Myint, Than-Aye. Farmers’ knowledge, attitudes, and practices for rodent management in Myanmar. Intl J Pest Manag. 2008;54(1):69–76.

[22] Meerburg BG, Singleton GR, Leirs H (2009). The year of the rat ends - time to fight hunger!. Pest Manag Sci..

[23] Singleton GR, Belmain S, Brown PR, Aplin K, Nyo Me H (2010). Impacts of rodent outbreaks on food security in Asia. Wildlife Res.

[24] Palis FG, Singleton G, Sumalde Z, Hossain M (2007). Social and cultural dimensions of rodent pest management. Integr Zool.

[25] Stenseth NC, Leirs H, Skonhoft A, Davis SA, Pech RP, Andreassen HP, Singleton GR, Lima M, Machang'u RS, Makundi RH, Zhang ZB, Brown PR, Shi DZ, Wan XR. Mice, rats, and people: the bio-economics of agricultural rodent pests. Front Ecol Environ. 2003;1(7):367–75.

[26] Meerburg BG, Singleton GR, Kijlstra A (2009). Rodent-borne diseases and their risks for public health. Crit Rev Microbiol.

[27] Stenseth NC, Atshabar BB, Begon M, Belmain SR, Bertherat E, Carniel E, Gage KL, Leirs H, Rahalison L (2008). Plague: past, present, and future. PLoS Med.

[28] Meerburg BG, Kijlstra A (2007). Role of rodents in transmission of *Salmonella* and *Campylobacter*. J Sci Food Agric.

[29] Pandey DP. Venomous snakes of medical relevance in Nepal: study on species, epidemiology of snake bite and assessment of risk factors of envenoming and death. Frankfurt: Goethe University; 2015. Available in: http://publikationen.ub.uni-frankfurt.de/frontdoor/index/index/docId/38272. Accessed 15 Dec 2016.

[30] Ceriaco LMP (2012). Human attitudes towards herpetofauna: the influence of folklore and negative values on the conservation of amphibians and reptiles in Portugal. J Ethnobiol Ethnomed..

[31] Ceriaco LMP, Marques MP, Madeira NC, Vila-Vicosa CM, Mendes P (2011). Folklore and traditional ecological knowledge of geckos in southern Portugal: implications for conservation and science. J Ethnobiol Ethnomed..

[32] Alves RRN, Vieira KS, Santana GG, Vieira WLS, Almeida WO, Souto WMS, Montenegro PFGP, Pezzuti JCB (2012). A review on human attitudes towards reptiles in Brazil. Environ Monit Assess.

[33] Frembgen JW (1996). The folklore of geckos: ethnographic data from south and west Asia. Asian Folkl Stud.

[34] Alves RRN, Filho GAP (2007). Commercialization and use of snakes in North and Northeastern Brazil: implications for conservation and management. Biodivers Conserv.

[35] Fita DS, Costa Neto EM, Schiavetti A (2010). ‘Offensive’ snakes: cultural beliefs and practices related to snakebites in a Brazilian rural settlement. J Ethnobiol Ethnomed..

[36] Shah KB (2001). Herpetofauna and ethnoherpetology of the southern Annapurna region, Kaski district, Nepal. J Nat Hist Mus..

[37] Flatt VB (2008). Act locally, affect globally: How changing social norms to influence the private sector shows a path to using local government to control environmental harms. Boston Coll Environ Aff Law Rev.

[38] King Mahendra Trust for Nature Conservation (1998). Buffer zone policy analysis of the Royal Chitwan National Park.

[39] Central Bureau of Statistics (2012). National population and housing census 2011: Village Development Committee/Municipality.

[40] United Nations Environment Programme. Royal Chitwan National Park. United Nations Environment Programme, World Conservation Monitoring Centre. 2011. http://www.unep-wcmc.org/medialibrary/2011/06/13/e26c7182/Royal%20Chitwan.pdf. Accessed 16 Dec 2012.

[41] Huntington HP (2000). Using traditional ecological knowledge in science: methods and applications. Ecol Appl.

[42] Albuquerque UP (2014). Cunha, LVFC, Lucena, RFP, Alves, RRN 2014. Methods and techniques in ethnobiology and ethnoecology.

[43] Pandey DP (2012). Snakes in the vicinity of Chitwan National Park, Nepal. Herpetol Conserv Biol..

[44] World Health Organization (2010). Guidelines for the management of snakebites.

[45] Pandey DP, Thapa CL (2010). Recognition of venomous snakes, prevention, first aid treatment and misconceptions on snakes and snakebites (Nepali medium).

[46] Pandey DP, Khanal BP (2013). Inclusion of incorrect information on snakebite first aid in school and university teaching materials in Nepal. J Toxicol Environ Health Sci.

[47] Shah KB, Sherstha JM, Thapa CL (2003). Snakebite management guideline.

[48] Sutherland SK, Coulter AR, Harris RD (1979). Rationalisation of first aid measures for elapid snakebite. The Lancet.

[49] Tun-Pe, Aye-Aye-Myint, Khin-Ei-Han, Thi-Ha, Tin-Nu-Swe. Local compression pads as a first aid measure for victims of bites by Russell’s Viper (*Daboia russelii siamensis*) in Myanmar. Trans R Soc Trop Med Hyg. 1995;89(3):293–95. 10.1016/0035-9203(95)90547-2 7660439

[50] Wilcoxon F (1945). Individual comparisons by ranking methods. Biometrics Bull..

[51] Zug GR, Mitchell JC (1995). Amphibians and reptiles of the Royal Chitwan National Park, Nepal. Asiat Herpetol Res..

[52] Chettri B, Bhupathy S (2007). Reptile fauna of Sikkim with emphasis to the Teesta valley. J Hill Res.

[53] Alves RRN, Silva VN, Trovao DMBM, Oliveira JV, Mourao JS, Dias TLP, Alves AGC, Lucena RFP, Barboza RRD, Montenegro PFGP, Vieira WLS, Souto WMS. Students’ attitudes toward and knowledge about snakes in the semiarid region of northeastern Brazil. J Ethnobiol Ethnomed. 2014;10:30. 10.1186/1746-4269-10-30 PMC3986856 24673877

[54] Wojnowski D (2009). Scientific and traditional conceptions of snakes in Kenya: herpetologists as teacher mentors. Herpetol Rev.

[55] Reading CJ, Luiselli LM, Akani GC, Bonnet X, Amori G, Ballouard JM, Filippi E, Naulleau G, Pearson D, Rugiero L. Are snake populations in widespread decline? Biol Lett. 2010;6(6):777–80. 10.1098/rsbl.2010.0373 PMC3001371 20534600

[56] Thomas CD, Cameron A, Green RE, Bakkenes M, Beaumont LJ, Collingham YC, Erasmus BF, De Siqueira MF, Grainger A, Hannah L. Extinction risk from climate change. Nature. 2004;427(6970):145–48. 10.1038/nature02121 14712274

[57] Chapin III FS, Zavaleta ES, Eviner VT, Naylor RL, Vitousek PM, Reynolds HL, Hooper DU, Lavorel S, Sala OE, Hobbie SE, Mack MC, Diaz S. Consequences of changing biodiversity. Nature. 2000;405(6783):234–42. 10.1038/35012241 10821284

[58] Chhettri K, Chhetry DT (2013). Diversity of snakes in Sarlahi District, Nepal. Our Nature..

[59] Nepal Bible Society (2003). The holy bible. Kishor Offset Press (P.), Ltd..

[60] Linnaeus C. Systema naturae per regna tria naturae, secundum classes, ordines, genera, species, cum characteribus, differentiis, synonymis, locis. 10th ed. Stockholm, Sweden: Impensis L. Salvii; 1758.

[61] Burghardt GM, Murphy JB, Chiszar D, Hutchins M, Mullin SJ, Seigel RA (2009). Combating ophiophobia: origins, treatment, education, and conservation tools. Snakes: ecology and conservation.

[62] Dodd CK, Seigel RA, Collins JT, Novak SS (1987). Status, conservation, and management. Snakes: ecology and evolutionary biology.

[63] Ohman A, Mineka S (2001). Fears, phobias, and preparedness: toward an evolved module of fear and fear learning. Psychol Rev.

[64] Isbell LA (2006). Snakes as agents of evolutionary change in primate brains. J Hum Evol.

[65] Sharma SK, Chappuis F, Jha N, Bovier PA, Loutan L, Koirala S (2004). Impact of snakebites and determinants of fatal outcomes in southeastern Nepal. Am J Trop Med Hyg.

[66] Pandey DP (2007). Epidemiology of snakebites based on field survey in Chitwan and Nawalparasi Districts, Nepal. J Med Toxicol..

[67] Shah KB, Schleich HH, Kästle W (2002). Amphibians and reptiles in Nepalese culture and economy. Amphibians and reptiles of Nepal: biology, systematics, field guide.

[68] Zhou Z, Jiang Z (2004). International trade status and crisis for snake species in China. Conserv Biol.

[69] Alves RRN, Filho GAP, Delima YCC (2007). Snakes used in ethnomedicine in northeast Brazil. Environ Devel Sustainability.

[70] Maskey T, Schleich HH, Kästle W, Schleich HH, Kästle W (2002). Nepal’s herpetofauna on a razor’s edge between threat and conservation. Amphibians and reptiles of Nepal: biology, systematics, field guide.

[71] Joshi T, Joshi M (2010). Ethno-ophiology: a traditional knowledge among tribes and non-tribes of Bastar, Chhattisgarh. IJTK..

[72] Silva A, Gamlaksha D, Waidyaratne D (2013). Medico-legal significance of the identification of offending snake in a fatal snake bite: a case report. J Forensic Leg Med.

[73] Corbett SW, Anderson B, Nelson B, Bush S, Hayes WK, Cardwell MD (2005). Most lay people can correctly identify indigenous venomous snakes. Am J Emerg Med.

[74] Werner D, Thuman C, Maxwell J (2011). Where there is no doctor: a village health care handbook.

[75] Suchard JR, LoVecchio F (1999). Envenomations by rattlesnakes thought to be dead. New England J Med.

[76] Griffen D, Donovan JW (1986). Significant envenomation from a preserved rattlesnake head (in a patient with a history of immediate hypersensitivity to antivenin). Ann Emerg Med.

[77] Keyler DE, Schwitzer K (1987). Envenomation from the fang of a freeze-dried prairie rattlesnake head. Vet Hum Toxicol.

[78] Pandey DP (2006). Epidemiology of snakebite based on hospital survey in Chitwan and Nawalparasi Districts, Nepal. J Nepal Health Res Counc..

[79] Pandey DP, Thapa CL, Hamal PK (2010). Impact of first aid training in management of snakebite victims in Madi Valley. J Nepal Health Res Counc.

[80] Rahman R, Faiz MA, Selim S, Rahman B, Basher A, Jones A, d'Este C, Hossain M, Islam Z, Ahmed H, Milton AH. Annual incidence of snakebite in rural Bangladesh. PLoS Negl Trop Dis. 2010;4(10):e860. doi:10.1371/journal.pntd.0000860. 10.1371/journal.pntd.0000860 PMC2964284 21049056

[81] Chandio AM, Sandelo P, Rahu AA, Ahmed ST, Dahri AH, Bhatti R (2000). Snakebite: treatment seeking behaviour among Sindh rural population. J Ayub Med Coll.

[82] Inamdar IF, Aswar NR, Ubaidulla M, Dalvi SD (2010). Snakebite: admissions at a tertiary health care center in Maharashtra, India. S Afr Med J..

[83] Magar CL, Devkota K, Gupta R, Shrestha RK, Sharma SK, Pandey DP (2013). A hospital based epidemiological study of snakebite in Western Development Region, Nepal. Toxicon..

[84] Pandey DP, Vohra R, Stalcup P, Shrestha BR (2016). A season of snakebite envenomation: presentation patterns, timing of care, anti-venom use, and case fatality from a hospital of southcentral Nepal. J Venom Res..

[85] Pandey DP, Thapa CL (2010). Snake and human life: a positive quest (Nepali medium).

[86] Miranda TM, De Mello Amorozo MC, Govone JS, Miranda DM (2007). The influence of visual stimuli in ethnobotanical data collection using the listing task method. Field Methods.

[87] Alexiades MN, Sheldon JW (1996). Selected guidelines for ethnobotanical research: a field manual.

